# Above- and belowground succession following multiple-tree mortality in *Pinus densiflora* forests

**DOI:** 10.3389/fpls.2025.1737673

**Published:** 2026-01-22

**Authors:** Seung–Jae Lee, Ah–Rim Lee, Dong–Seok Son, Dong–Bin Shin, Seok Hui Lee, Yeong-Eun Kim, Jun Won Kang, Seung–Hwan Oh

**Affiliations:** 1 Department of Forestry, The Graduate School of Kyungpook National University, Daegu, Republic of Korea; 2 Wetland Center, National Institute of Ecology, Changnyeong, Republic of Korea; 3 School of Forest Sciences and Landscape Architecture, Kyungpook National University, Daegu, Republic of Korea

**Keywords:** climate change, coarse woody debris, microbial communities, *Pinus densiflora*, rhizosphere soil, vegetation succession

## Abstract

Climate-driven tree mortality impacts forest ecosystems, yet studies rarely integrate above- and below-ground processes. Consequently, the temporal dynamics of rhizosphere microbial communities following mortality and their link to vegetation changes remain unclear. This study examined coupled shifts in species composition and rhizosphere microbial communities following multiple-tree mortality events in *Pinus densiflora* stands. We employed a chronosequence approach by reconstructing tree mortality timing and analyzing temporal changes in above-ground species composition alongside rhizosphere microbial diversity. Random Forest (RF) traced microbial community transitions from healthy to declining and dead trees, identifying core genera indicative of tree health, followed by an analysis of co-occurrence network dynamics along the health–decline–mortality sequence. As a result of this study: (1) Despite increased light from canopy decay, understory changes remained subtle. However, 15 years post-mortality, snag collapse expanded gaps, promoting active *P. densiflora* regeneration and signaling potential recovery. (2) Rhizosphere microbial α-diversity in healthy trees was lower or similar compared to declining and dead trees. (3) RF analysis identified the abundance of specific microbial groups—notably *Oidiodendron* spp. (Ascomycota) and *Umbelopsis* spp. (Mucoromycota)—as a key indicator of healthy *P. densiflora* forests. This finding suggests that specific functional microbial composition (key microbial groups associated with tree health), rather than overall diversity, is a critical factor for tree health, which is supported by our α–diversity results showing that healthy forests had lower diversity than declined forests. (4) In fungal communities, compared to healthy *P. densiflora* stands, declining and recently dead stands (3 years post-mortality) formed denser and more complex networks (more nodes and edges). Notably, this complexity was characterized by significantly lower modularity within the largest connected component, despite exhibiting similar or higher whole-network modularity. In contrast, networks at the oldest mortality sites (~15 years) became smaller and simpler, with similar modularity and resembling healthy stands at the whole-network level. By examining temporal above- and below-ground changes, this study provides insights into forest regeneration processes, emphasizing above-ground–below-ground interactive dynamics and highlighting that the abundance of specific indicator microbial groups, rather than overall microbial diversity, plays a more critical role in assessing tree health.

## 1 Introduction

Ecosystems worldwide are directly affected by climate change, and the decline of coniferous forests is being accelerated by interactions between complex factors, such as elevated temperatures and water stress (Bréda et al., 2006; Allen et al., 2010). Many recent studies have focused on coniferous forests in alpine and sub-alpine regions, which are vulnerable to environmental changes, and efforts have been directed toward understanding the structural changes and ecological mechanisms in these forests (Hock et al., 2019; Verrall and Pickering, 2020; Lee et al., 2023b). This is because these regions are strongly influenced by climatic conditions and respond sensitively to sudden environmental changes, including rising temperatures, changes in rainfall patterns, and habitat shrinkage (Engler et al., 2011; Guisan et al., 2019; Lee et al., 2025). Meanwhile, research on the effects of climate change on lowland coniferous forests is relatively lacking, and this could be because, unlike in alpine ecosystems, the process of decline due to climate change in these forests is not immediate or obvious.

*Pinus densiflora* Siebold & Zucc, which is widely distributed throughout East Asia, is a well-established temperate conifer that grows at relatively low altitudes (Nakamura and Krestov, 2005) and exhibits strong adaptability to drought and barrenness (Lee et al., 2018). Recently, however, in South Korea, as physiological stress has been increasing due to climate change, multiple-tree mortality events have been persistently reported (Kim et al., 2020; Lee et al., 2023a). Furthermore, despite the tolerance of the species, increasing temperature and changes in rainfall patterns are increasingly considerably affecting *P. densiflora* habitats, increasing the possibility of habitat change and structural decline (Chun and Lee, 2013; Duan et al., 2022). *P. densiflora* occupies a broad area throughout East Asia, and the protection of these forests is viewed as an important factor contributing to the formation of mixed forest ecosystems co-inhabited by broadleaf and coniferous trees. Understanding stand dynamics driven by this species’ physiological responses to climate change—including changes in above-ground species composition and below-ground rhizosphere soil dynamics—and tracking shifts in indicator microbial communities that provide early warnings of tree decline are essential for safeguarding the long-term stability of temperate forest ecosystems and for designing sustainable management plans. Such an integrated perspective will improve our understanding of *P. densiflora* forest behavior and provide valuable reference data for evaluating the regeneration potential and ecological succession processes of lowland coniferous forests under climate change. For example, forest gaps that form due to the death of overstory vegetation alter the availability of light within the stand (Heinrichs and Schmidt, 2009; Lee et al., 2024). This promotes the entry and establishment of understory vegetation, providing important clues to help understand mechanisms of species regeneration and changes in stand behavior. In particular, in order to understand the nature of changes in *P. densiflora* forests, analyzing the species that become established after the decline of the previously dominant species, whether *P. densiflora* is re-established, and the type of environmental conditions that support re-establishment is important. In addition, the rhizosphere is one of the most complex and sensitive areas, affected by plant roots and numerous soil microbial communities (Egamberdieva et al., 2008; De Feudis et al., 2017). It also plays important roles in plant nutrition and adaptation to various types of stress (Kumawat et al., 2022). Soil microbial communities that interact with plants provide various adaptive benefits, such as promoting the growth of the host, improving nutrient absorption, and enhancing stress tolerance (Dubey et al., 2019; Banerjee and van der Heijden, 2023). However, previous studies on stand regeneration have mostly focused on either above- or belowground components, and the temporal changes in belowground microbial communities are yet to be quantitatively analyzed (e.g., changes in microbial communities after tree decline).

In this study, we focused on sites of multiple-tree mortality of *P. densiflora* in South Korea. We defined multiple-tree mortality as cases involving at least four dead canopy layer (overstory) trees within a 20-m radius of a standing dead *P. densiflora* tree, attributing the deaths to shared local environmental conditions affecting the initial dead tree. This specification was necessary because tree death can be caused by various factors such as competition or individual characteristics (Kim and Lee, 2023). Therefore, the causes of single-tree mortality may differ from those of multiple-tree mortality. In addition, analyzing sites of multiple-tree mortality can reflect the cumulative effects of climate change or other environmental stressors that are difficult to identify from case studies of individual tree deaths. Thus, we expected this approach to effectively reveal stand-level responses to disturbance. To elucidate how both above-ground vegetation dynamics and below-ground rhizosphere processes—including early-warning shifts in indicator microbial communities that accompany the initial stages of tree decline—shape post-mortality succession in *P. densiflora* stands, we formulated the following hypotheses:

(a) We hypothesized that patterns of understory cover and species composition, including the regeneration of *P. densiflora*, would be shaped by interactions between time since tree death and forest-floor substrate characteristics (e.g., rock exposure and bare soil). (b) Predicted that healthy *P densiflora* would show changes in the microbial community composition depending on the decay class with time after decline and tree death. We hypothesized that it would be possible to identify specific microbes that are more abundant in the healthy state. (c) As decomposition proceeds after tree death (with decay class reflecting time since death), fungal–bacterial co-occurrence networks characteristic of healthy *P. densiflora* stands—dense and complex—will progressively transition toward sparser, less cohesive, and functionally reassembled configurations. By integrating analyses of aboveground vegetation succession and belowground microbial community shifts, this study aims to elucidate the ecological changes occurring in declining *P. densiflora* forests. We anticipate that the results will provide baseline information for evaluating regeneration potential and the sustainability of lowland coniferous forests under climate change and will inform targeted restoration and management strategies.

## 2 Materials and methods

### 2.1 Study area, remote sensing survey and division of mortality time series

**Figure 1:**
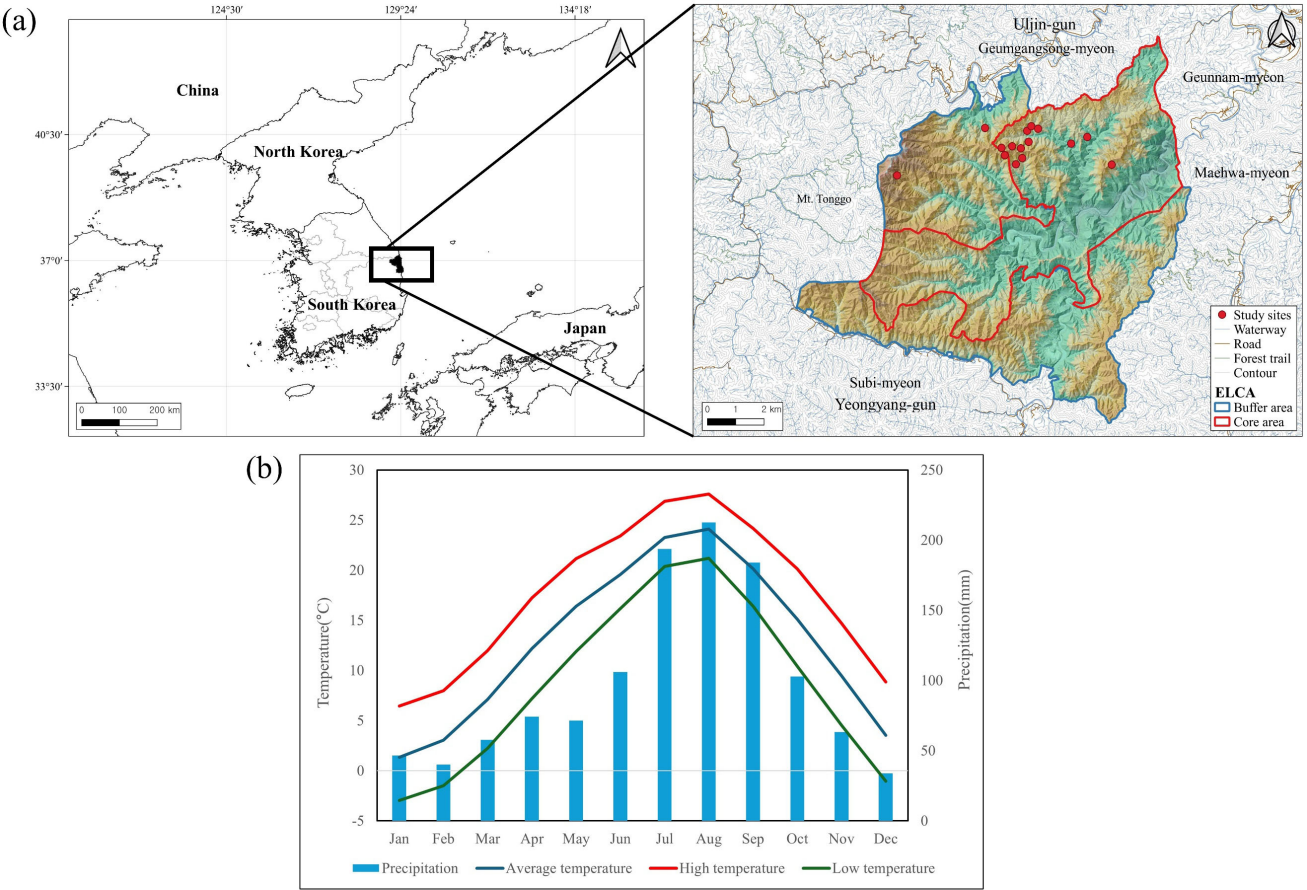
Geographical locations and climatic characteristics of South Korea and the Wangpicheon Ecosystem and Landscape Conservation Area (ELCA). **(a)** Geographic locations and topography of 15 P*. densiflora* mass mortality sites within the ELCA. **(b)** Mean temperature and precipitation data from 1990–2023 based on meteorological records from the Ulsin region, approximately 20 km from the ELCA.

The study area was the Wangpicheon Ecosystem and Landscape Conservation Area (ELCA), located in central South Korea. This region was designated by the Ministry of Environment in 2005 as a protected area for habitat conservation for endangered species and rare wild animals and plants. The *P. densiflora* growing in this region are the largest in South Korea and grow with characteristically straight trunks. The total area of the conservation area is 102.84 km2, and according to forest type maps (1:5,000) from the Korea Forest Service, *P. densiflora* occupies the widest area, at approximately 35.37 km2 (34.40%; **Supplementary Table S1**). When we analyzed meteorological data for 1990–2023 using data from the Uljin Meteorological Office, the mean annual temperature was 12.9 °C and the mean annual rainfall was 1,186.64 mm, with most rainfall concentrated in the period from July to September (**Figure 1b**). To identify sites of multiple-tree mortality of *P. densiflora* within the conservation area, we conducted remote sensing using drones over 3 years from 2021 to 2023. Using the collected orthophotos (resolution 5–10 cm), we identified dead *P. densiflora* based on information such as color and appearance (Kim J. et al., 2017) and then extracted multiple-tree *P. densiflora* mortality sites based on the definition of these sites (**Supplementary Figure S1a**). Later, we conducted field surveys to verify the multiple-tree mortality of *P. densiflora* at these locations. Using these methods, we were ultimately able to identify 15 areas where multiple-tree mortality had occurred (**Figure 1a**). Due to the strict management system in this region, pine wilt disease and other pests are absent. When comparing climate conditions during the study period (1990–2023; **Figure 1b**) with historical climate data prior to 1990(**Supplementary Figure S2**), we attributed the observed *P. densiflora* mortality primarily to physiological stress induced by recent climatic changes, including more frequent and prolonged droughts, elevated temperatures, and altered precipitation patterns (e.g., decreased precipitation in winter and spring). Here, we defined climate change-related mortality as multiple-tree mortality occurring in the absence of pest or disease infestation and temporally coinciding with periods characterized by these documented climatic stressors. Indeed, no pest damage was observed during our field surveys.

Next, using the 15 secured multiple-tree mortality sites, we analyzed existing aerial photographs to identify the time of tree death and to estimate the time since tree death. For this purpose, we used aerial photographs from the South Korean National Geographic Information Institute, Kakao Map, and Google Earth Pro. From the images, we estimated the time of tree death to be the time when the tree branches first spread in a star shape, and the color began to change to white or red. By analyzing previous aerial images, we broadly categorized multiple-tree mortality of *P. densiflora* in the survey area into three stages (**Supplementary Figures S4**, **S5**): tree deaths before 2008 or from 2008 to 2011 were classified as Group 1 (n = 6). These were the oldest cases in this study, and as of 2024, over 15 years have passed since the multiple-tree mortality event. Tree deaths occurring from 2014 to 2017 were classified as Group 2 (n = 7). These were defined as intermediate multiple-tree mortality events that occurred 7–10 years ago, as of 2024. Finally, tree deaths occurring from 2020 to 2021 were classified as Group 3 (n = 2). These were the most recent multiple-tree mortality events, occurring approximately 3 years before the study period in 2024.

### 2.2 Vegetation survey and soil physicochemical analysis

We conducted field surveys of the 15 P*. densiflora* multiple-tree mortality sites during the active tree-growth period from June to September 2024. We also selected and surveyed control plots comprising healthy *P. densiflora* forests with similar local conditions located at least 30 m from the multiple-tree mortality sites and, owing to the intervening topography and slope, positioned so that dead or declining trees were not visible from the control plots. Therefore, we conducted field surveys at 30 sites in total. Considering the local stand characteristics and number of dead trees, we used survey plot sizes of 20 m × 10 m (12 sites), 15 m × 15 m (1 site), 20 m × 20 m (1 site), and 20 m × 15 m (1 site) for the tree mortality sites. For all control sites, we conducted the surveys within 20 m × 10 m quadrats. For the vegetation survey, we investigated dominance, combining the coverage and abundance of species in the plots by stratum (tree, sub-tree, shrub layer, and herb layers) in accordance with the phytosociological survey method of Braun-Blanquet (1964). The dominance index of each species was determined using the Braun-Blanquet scale as follows: r, rare species; +, coverage <1%; 1, coverage 1–5%; 2, coverage >5–25%; 3, coverage >25–50%; 4, coverage >50–75%; 5, coverage >75–100% (Canullo et al., 2012). Diameter at breast height (DBH) was surveyed for all individuals in the plot with a DBH of at least 6 cm. For environmental factors, we used a handheld GPS 64S (Garmin, Olathe, KS, USA) and clinometer (Suunto, Vantaa, Finland) to record the elevation, slope, slope direction, and geographic coordinates, and we also recorded rock exposure and topographic position in the field. For *P. densiflora* with a DBH of <6 cm, we surveyed three different tree height categories (h ≤ 10 cm, 10 < h < 50 cm, h ≥ 50 cm) using the folding rule. Next, we evaluated the decay class of coarse woody debris (CWD) from *P. densiflora* in the plots, surveying snag (standing dead trees), downed wood (i.e., logs), and stumps separately. To evaluate the decay class of snag and log, we referred to the stand-level biodiversity monitoring protocol steps for field data collection from British Columbia (Province of British Columbia, 2009; **Supplementary Figure S3**), and for stumps we referred to data from Rouvinen and Kouki (2002; **Supplementary Table S2**). Finally, we used a fisheye lens at the center of each multiple-tree mortality survey plot to obtain crown projection data and analyze crown openness. Crown openness was analyzed using Gap Light Analyzer 2.0 (Frazer et al., 1999). The multiple-tree mortality sites have been summarized in **Supplementary Table S3**.

For soil physicochemical analysis, three bulk soil samples were taken from a depth of 0–10 cm within a 1-m radius of dead trees and mixed after removing the organic horizon at multiple-tree mortality site. In the control plots, soil samples were collected within a 1-m radius of living *P. densiflora* trees and assessed using the same method as for the multiple-tree mortality sites. We collected 15 samples each from multiple-tree mortality sites and control plots and transferred the samples to a laboratory where they were naturally dried, passed through a 2-mm sieve, and sealed. The samples were then sent to the South Korean Analysis Technology Center, a professional soil sample analysis company. The methods of analysis for each sample are described in **Supplementary Table S4**. In summary, we analyzed 11 chemical properties, including total nitrogen (N), exchangeable cations (K^+^, Ca²^+^, Mg²^+^, and Na^+^), available phosphorus (measured as Olsen-P), organic carbon (C), organic matter (OM), cation exchange capacity (CEC), soil pH, and electrical conductivity (EC).

### 2.3 Rhizosphere soil sampling, sequencing, and bioinformatic processing

For the rhizosphere soil survey, we combined analysis of past aerial photographs from multiple-tree mortality sites identified using prior remote sensing with the results of decay class assessment conducted in the field. Based on the results, we collected samples according to the time since tree death. We aimed to minimize errors due to local conditions by collecting samples from nearby survey sites in locations with similar conditions; the selected sites were located in close proximity to each other and shared similar ridge-top soil properties, microclimatic conditions (e.g., strong winds and ample sunlight), and initial stand structure. Therefore, among the total 15 multiple-tree mortality sites, we selected 10 representative plots that best met these strict homogeneity criteria for the final analysis in Section 2.4. To minimize micro-scale soil heterogeneity, three subsamples were collected from different directions around the rhizosphere of each individual tree and pooled into a single composite sample. Specifically, we collected soil tightly adhering to fine roots, which distinguishes these samples from the bulk soil collected for physicochemical analysis in Section 2.2. For Group 1 we selected three snags (decay class 6) each from three sites and collected soil samples from depths of 0–10 cm after removing the organic horizon. For Group 2 we selected three snags (decay class 4) each from five sites for soil collection. For Group 3 we selected three snags (decay class 3) each from two sites for sample collection. Of these, one site contained decay class 3 dead trees (twigs and pinecones remaining, showing the same decay condition as a multiple-tree mortality site from 3 years prior) located at the site of a previous multiple-tree mortality site from 2008. To compare with sites of tree mortality, we collected control rhizosphere soil samples from healthy *P. densiflora* forests in similar locations at least 50 m from the soil samples collected from multiple-tree mortality sites. In this process, we selected three healthy *P. densiflora* at each of the four sites and collected samples from locations where dead trees were not visible due to the terrain and slope, to ensure that the sampling was not influenced by the extending root systems or mycelia of nearby dead trees. We additionally collected four rhizosphere soil samples from one declining *P. densiflora* forest site (defined as the ‘decline’ group). Here, the health condition of the declining trees was evaluated based on defoliation in accordance with the method of Huo et al. (2019). The trees were found to be in the 40–80% defoliation range (**Supplementary Figure S6**). All the rhizosphere soil samples were placed in 50-mL conical tubes and transported to the laboratory in an icebox to maintain a low temperature. At the laboratory, the samples were stored at -20 °C. The local conditions for all collected samples showed the characteristics of a ridge environment, with strong winds and ample sunlight. The local conditions and soil environment at the collection sites are described in **Supplementary Table S5**.

Subsequently, after transporting the soil samples to the laboratory at a low temperature, a DNeasy^®^ PowerSoil^®^ Pro Kit (Qiagen, Hilden, Germany) was used to extract DNA from the stored samples, and a Nanodrop (ND-LITE-PR; Thermo Fisher Scientific, Waltham, MA, USA) was used for quality control. DNA sequencing was performed at the Kyungpook National University NGS Center (Daegu, Republic of Korea). A Miseq system (Illumina, San Diego, CA, USA) was used for bacterial 16S V4 amplicon sequencing, and a Hiseq3000 (Illumina, San Diego, CA, USA) was used for fungal ITS2 amplicon sequencing. The amplified DNA set was cleaned using Beckman Coulter AMPure XP (Thermo Fisher Scientific, Waltham, MA, USA) and amplified again to tag the different barcodes of each sample. The completed libraries were sequenced using each Illumina platforms using universal primer pairs (515F/805R and ITS86F/ITS4R) (**Supplementary Table S6**).

Finally, we used QIIME2 and DADA2 to remove chimera reads, compile phylogenetic trees, and perform classification and normalization from the DNA raw data (Callahan et al., 2016; Bolyen et al., 2019). The databases used for classification included SILVA (v 138) for bacteria and UNITE (v 8.3) for fungi (Abarenkov et al., 2010; Bokulich et al., 2018). To compile phylogenetic trees, we used SATé-enabled phylogenetic placement (SEPP) and the SILVA database for bacteria and Mafft 7 for fungi (Mirarab et al., 2012; Katoh and Standley, 2013). Furthermore, all samples were rarefied to an equal sequencing depth, and the rarefied feature tables were used for downstream statistical analysis and visualization (**Supplementary Table S7**).

### 2.4 Data analysis

#### 2.4.1 Analysis of CWD dynamics and *P. densiflora* regeneration

Based on the results of the CWD decay evaluation for the 15 multiple-tree mortality sites collected in the field, we plotted the distributions of data depending on the time of tree death (categorized as Group 1–3) for each type (snag, log, stump). Here, by examining how the CWD decay process changes over time and analyzing the distribution properties by CWD type (snag, log, stump) over time, we aimed to compare whether forest structural changes (e.g., forest gaps) and the estimated time of tree death at each multiple-tree mortality site were consistent with changes in local conditions. We also analyzed the distribution of *P. densiflora* saplings by tree height alongside changes with time since tree death to evaluate whether continual regeneration occurred after *P. densiflora* mortality. These distributions were visualized using the ggplot2 package in R (Wickham et al., 2016).

#### 2.4.2 Analysis of relationships among species, soil, and environmental variables

We performed canonical correspondence analysis (CCA) using the vegan package (Oksanen et al., 2025) to examine changes in aboveground species composition with time since tree death at multiple-tree mortality sites and compared site characteristics between mortality and control sites. CCA is useful for analyzing relationships between environmental variables and species and determining how species distribution patterns could be explained by environmental variables. In our analysis, we used soil chemistry characteristics, species richness (SR), and the total coverage of the 16 most frequent species in the understory (shrub and herb layers) of the control and multiple-tree mortality sites. To address potential sampling bias due to varying plot sizes (200–400 m²), we evaluated whether differences in sampling area influenced species richness. We fitted a linear regression of species richness against plot area and performed a one-way ANOVA with plot area treated as a categorical factor; neither analysis revealed a significant effect of plot size on species richness (**Supplementary Table S8**). Therefore, we used species richness per plot without further area standardization in subsequent analyses. For the vegetation dataset, we used a modified version of Hellinger’s method (Rao, 1995). We also included locational properties of elevation, rock exposure, and slope in the analysis, and we calculated the basal area (BA) of Quercus spp. and *P. densiflora* growing within the survey plots. In the CCA between multiple-tree mortality sites, we also included canopy openness, calculated from images obtained using a fisheye lens. We applied a log transformation to the environmental variables used in the analysis to improve their linearity and normality. We calculated variance inflation factors (VIFs) to identify multicollinearity. After removing predictive variables with high VIF values, we re-calculated VIFs for the remaining variables and repeated the process until all variables had a VIF <5. These variables were selected for the final analysis. Furthermore, we used 999 permutation tests to statistically assess the CCA (overall model, individual axes) and to identify which variables had a statistically significant influence (marginal and sequential). Lastly, we employed univariate linear regression and Spearman rank correlation analyses to statistically examine and clarify specific pairwise relationships suggested by the visual patterns in the CCA ordination diagram. To provide more detailed information about species growing after multiple-tree mortality of *P. densiflora*, we calculated importance values (IVs) using the method of Curtis and McIntosh (1951). IVs were calculated as the average after adding the relative frequency and the relative coverage. The ‘Decline’ group was excluded from this plot-level analysis because it consisted of a single survey site, which was insufficient for robust multivariate comparison.

#### 2.4.3 Microbial data statistical analysis

For α-diversity analysis, we divided the samples into five groups (Healthy, Groups 1–3, Decline) and calculated the Shannon (H′) and Chao1 diversity indices using the ‘vegan’ package (Oksanen et al., 2025). The Shannon index was calculated following Shannon (1948) (Equation 1), where *p*_i_ is the proportion of individuals belonging to the *i*-th species and *S* is the total number of species. The Chao1 species richness estimator was calculated following Chao (1984) (Equation 2), where *S_obs_* is the observed number of species, *F*_1_ is the number of singletons (species represented by a single individual), and *F*_2_ is the number of doubletons (species represented by two individuals). Differences in these indices among the groups were analyzed using one-way ANOVA with a Bonferroni *post-hoc* test. Non-metric multidimensional scaling (NMDS) was applied based on Bray–Curtis dissimilarity (Bray and Curtis, 1957; Kruskal, 1964) to visualize differences in bacterial and fungal communities between the five groups. The statistical significance of differences in community composition between the groups was tested using an analysis of similarities (ANOSIM) with 999 permutations (Clarke, 1993). The ‘vegan’ package (Oksanen et al., 2025) was used for the analysis. Next, we used the ‘randomForest’ R package (Liaw and Wiener, 2002) to identify indicator taxa determining differences in bacterial and fungal communities between the five groups (Healthy, Groups 1–3, Decline) and evaluate their importance. Before random forest (RF) analysis, we used the upSample function from the caret package (Kuhn, 2008) to correct imbalances in the sample size between groups and amplify the number of samples in each group. Next, we used the RF model to tune hyperparameters separately for the bacterial and fungal data. For the bacterial data, we randomly sampled 40 mtry values between 1 and 8 and used 40 ntree values at intervals of 100 in the range of 500 to 1,500. For the fungal data, we randomly sampled 20 mtry values between 1 and 6 and used 10 ntree values at intervals of 100 in the range of 500 to 1,500. For model validation, we determined the optimal hyperparameters based on the out-of-bag (OOB) errors (**Supplementary Figure S7**, **Supplementary Table S9**). Next, based on the optimized hyperparameters, we split the bacterial and fungal datasets into training (80%) and test (20%) data and performed 10-fold cross-validation. We compared the OOB error rates of the training and test data, validated the generalization performance of the model, and assessed the risk of overfitting by verifying that the difference in OOB error rates was not >0.15. The RF algorithm identified the main genera that showed high importance based on the mean decrease in accuracy between the five groups for bacterial and fungal data. All statistical analyses were conducted in R (R Core Team, 2025).

(1) $$H^{\prime }=-\sum_{i=1}^{S}p_{i}\ln(p_{i})$$

(2) $$S_{Chao1}=S_{obs}+\frac{F_{1}^{2}}{2F_{2}}$$

#### 2.4.4 Network construction

To investigate the relationships between each taxon in the taxa table obtained from amplicon sequencing, we constructed co-occurrence networks of bacteria and fungi. For each type (bacteria/fungi), we used genus-level data for the analysis. To ensure the robustness of the network analysis and effectively distinguish core taxa from transient species or sequencing noise, we applied a prevalence-based filtering approach. Specifically, we first converted the genus-level count data to relative abundances and then filtered taxa according to their prevalence across samples, retaining only bacterial genera present in more than 20% of samples and fungal genera present in more than 12% of samples. We calculated Spearman’s correlation (r) and applied sparsification based on t-tests when constructing the network. To handle 0 values, we used multiplicative replacement, and we applied CLR transformation to normalize the data. We set a criterion of r > 0.6 for the correlation coefficients and applied a significance level of p < 0.05. The p-values were adjusted using the Benjamini–Hochberg method. To ensure analysis stability, we also performed 1,000 bootstrap iterations (Efron and Tibshirani, 1994). To explore the modular structure within the networks, we applied the fast-greedy clustering algorithm proposed by Clauset et al. (2004). For network analysis, we constructed the networks using the ‘NetCoMi’ package (Peschel, 2025) in R, and we set the network layouts (circle) using the ‘igraph’ package (Csárdi and Nepusz, 2006).

## 3 Results

### 3.1 Temporal changes in *P. densiflora* deadwood and sapling distribution

**Figure 2:**
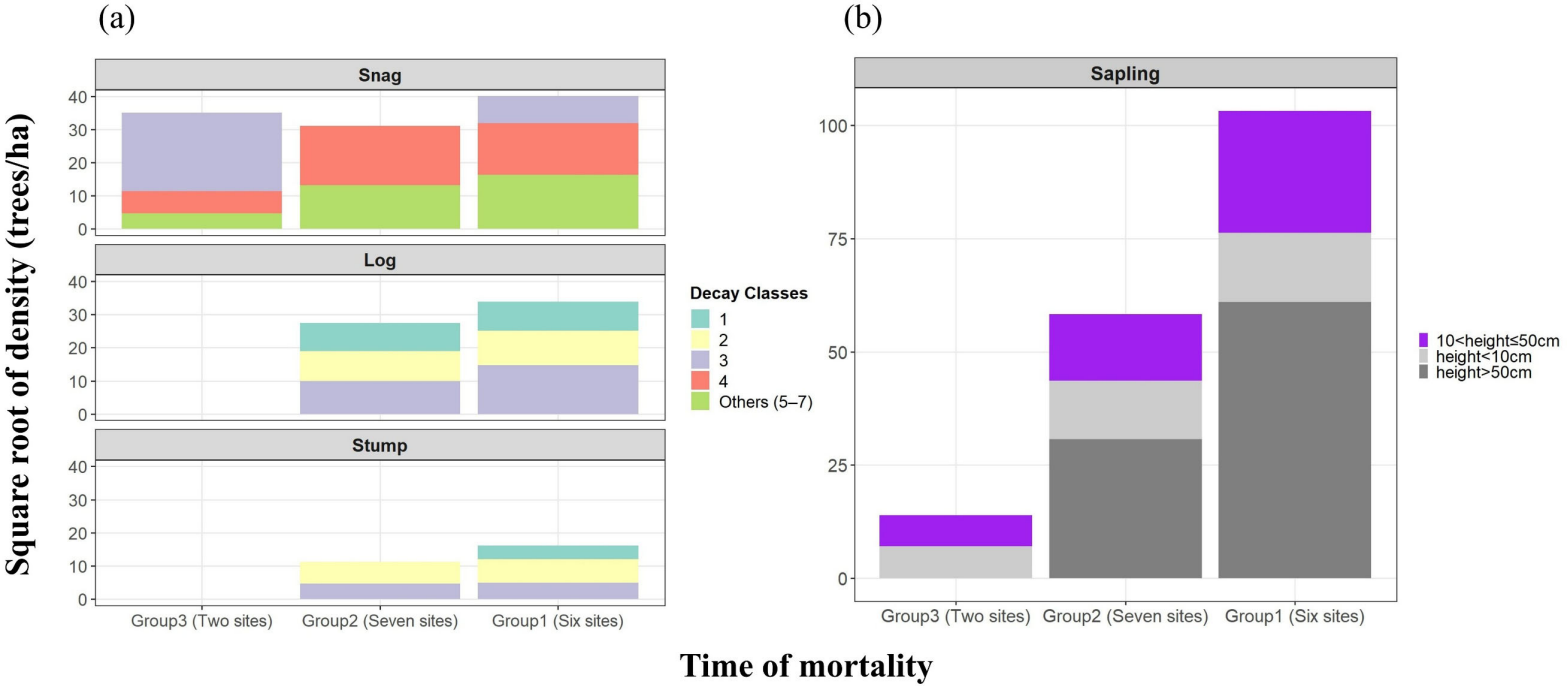
Distribution status of coarse woody debris (CWD) types classified by timing of mortality (reflecting differences in decay class) and height distribution of *P. densiflora* saplings based on data from 15 plots surveyed in the field. **(a)** shows the distribution status of CWD by timing of mortality, categorized into Snag, Log, and Stump, based on the decay class of deadwood. **(b)** shows the distribution of *P. densiflora* sapling regeneration by timing of mortality; saplings are defined as stems with DBH ≤ 6 cm and are further classified by height classes. The values represent per-hectare density (individuals ha^-^¹) averaged by group; a square-root transform was applied to reduce dispersion. Grades 5–7, which had low density, were combined into a single category and labeled as others.

*P. densiflora* CWD showed a gradual progression of decay from snags to logs and stumps (**Figure 2a**). Three years after tree death (Group3), the snags were mostly decay class 3, but after 7–10 years (Group2), they changed to decay class 4, and beyond 15 years (Group1), the snag forms reduced. *P. densiflora* saplings also showed almost no regeneration 3 years after tree death but regenerated as more time passed (**Figure 2b**). In addition, the extent of decay of some stumps and logs in Group 1 survey sites was so severe that it was difficult to evaluate the decay class; these were excluded from the analysis. Meanwhile, some snags observed in 2020–2021 had a relatively high decay class of 4–5, which was due to natural disasters (we observed burn marks consistent with lightning strikes).

### 3.2 CCA analysis and above-ground species traits

**Figure 3:**
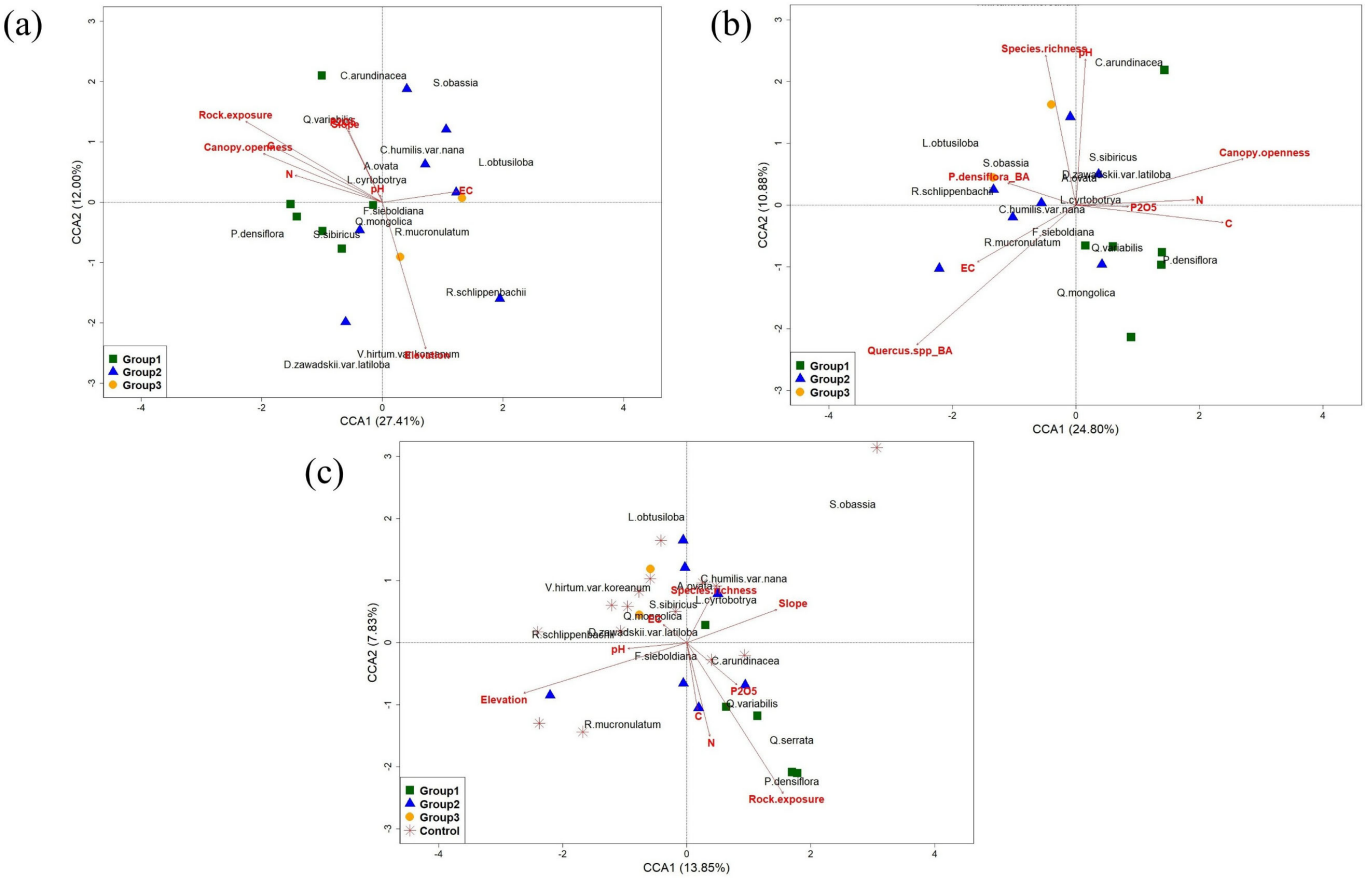
Canonical correspondence analysis (CCA) of understory vegetation in relation to topographic, vegetation, and soil chemical properties at multiple-tree mortality sites and control sites. **(a)** CCA showing the relationship between understory vegetation, topographic variables, and soil chemical properties across multiple-tree mortality sites, categorized by time since tree death. **(b)** CCA showing the relationship between understory vegetation, vegetation structural characteristics, and soil chemical properties across multiple-tree mortality sites, categorized by time since tree death. **(c)** CCA comparing understory vegetation, topographic variables, and soil chemical properties between multiple-tree mortality sites (grouped by time since tree death) and control sites. The abbreviations are as follows: total nitrogen (N), available phosphorus, organic carbon (C), organic matter (OM), cation exchange capacity (CEC), soil pH, and electrical conductivity (EC). Species BA refers to the basal area of the respective species. The full names of the 16 species used in the CCA analysis are listed in **Table 1** caption. To improve figure legibility, 1–2 species were omitted from **(a, b)** respectively, and one spatially isolated control plot was not displayed in **(c)**.

We performed CCA between multiple-tree mortality sites, differentiating by vegetation characteristics and local conditions. The analysis revealed no statistically significant environmental variables that distinguished the different multiple-tree mortality site groups from each other (**Figures 3a, b**; **Table 1**). In contrast, the analysis comparing all multiple-tree mortality sites against the control sites (**Figure 3c**) showed a significant separation between the two groups (Overall model p < 0.001). The key environmental variables driving this difference were pH, Elevation, and Rock exposure (**Table 1**). To summarize, compared with the other groups, Group 1 was strongly associated with *P. densiflora*, *Quercus*. spp, and *Rhododendron mucronulatum* in the understory vegetation, and strong associations with total nitrogen and available phosphorus, which indicate soil fertility in environments with open canopies and high rock exposure, were observed (**Figure 3**). Particularly, as shown in **Figure 3b**, canopy openness tended to be inversely related to *P. densiflora* BA and *Quercus* spp., suggesting that the local environment for Group 1 was characterized by open forest gaps following the death of overstory vegetation. To investigate this relationship in more detail, we performed univariate analyses between canopy openness and the basal area (BA) of *P. densiflora* and *Quercus* spp. The simple linear regression showed no significant association between canopy openness and the BA of either *P. densiflora* (Estimate = 0.001, p = 0.998) or Quercus spp. (Estimate = −0.794, p = 0.169). Similarly, the Spearman partial correlation analysis, revealed weak negative but non-significant correlations for both species (Estimate = −0.21, p = 0.59 for *P. densiflora*; Estimate = −0.19, p = 0.63 for *Quercus* spp.). When we compared characteristics between multiple-tree mortality and control sites, the control sites showed similar vegetation characteristics to Group 3 and some of Group 2. Specifically, compared with Group 1, these sites showed relatively high SR, strong associations with *Lindera obtusiloba* and *Vaccinium hirtum* var. *koreanum*, and were closely related to soil electrical conductivity and soil pH. The IVs analysis showed some differences in dominant species among the groups (**Table 2**). In Group 1 (the earliest mortality sites), *P. densiflora* was dominant. In contrast, *Fraxinus sieboldiana* was dominant in the intermediate (Group 2) and most recent (Group 3) mortality sites. Notably, Group 2 showed a relatively higher IV for *P. densiflora* compared to Group 3, and *P. densiflora* was not among the top 10 species in the Group 3 understory.

**Table 1:** Canonical correspondence analysis (CCA) of understory vegetation in relation to topographic, vegetation, and soil chemical properties at multiple-tree mortality sites and control sites.

Statistical analysis of the CCA results for Figure 3a										
Category		CCA1	CCA2	Variable		Marginal F (p)	Sequential F (p)	Overall model F (p)	Constrained R^2^	Adjusted R^2^
Axes	Eigenvalue (λ)	0.1753	0.0768	No statistically significant variables		–	–	1.168 (0.26)	0.678	0.101
	% constrained variance	27.41	12.00							
Statistical analysis of the CCA results for Figure 3b										
Axes	Eigenvalue (λ)	0.1586	0.0696	No statistically significant variables		–	–	0.994 (0.504)	0.641	0.006
	% constrained variance	24.80	10.88							
Statistical analysis of CCA results for Figure 3c										
Axes	Eigenvalue (λ)	0.1197	0.0677	Significant variables	pH	2.026 (0.034^*^)	1.905 (0.038^*^)	1.698 (0.001^***^)	0.433	0.182
	% constrained variance	13.85	7.83		Elevation	2.826 (0.002^**^)	3.418 (0.001^***^)			
					Rock exposure	2.240 (0.008^**^)	2.302 (0.013^*^)			

The table reports CCA1–2 eigenvalues and percent constrained variance. It also shows the statistics for the overall model (F-statistic, p-value, Constrained R², and Adjusted R²). The significance of each environmental variable was tested using both marginal (by = “margin”) and sequential (by = “term”) permutation tests (999 permutations). The full names of the 16 species used in the CCA analysis are listed: *Carex humilis* var. *nana*, *Atractylodes ovata*, *Pinus densiflora*, *Fraxinus sieboldiana*, *Quercus mongolica*, *Quercus variabilis*, *Quercus serrata*, *Rhododendron mucronulatum*, *Lespedeza cyrtobotrya*, *Spodiopogon sibiricus*, *Calamagrostis arundinacea*, *Rhododendron schlippenbachii*, *Dendranthema zawadskii* var. *latiloba*, *Styrax obassia*, *Lindera obtusiloba*, *Vaccinium hirtum* var *koreanum*.

**Table 2:** Status of understory vegetation importance values (IVs) for groups based on tree mortality timing.

Group1 (n = 5)		Group2 (n = 8)		Group3 (n = 2)	
Species	IVs	Species	IVs	Species	IVs
*Pinus densiflora* Siebold & Zucc.	20.45	*Fraxinus sieboldiana* Blume	20.27	*Fraxinus sieboldiana* Blume	16.00
*Fraxinus sieboldiana* Blume	15.67	*Carex humilis* Leyss. var. *nana* (H.Lév. & Vaniot) Ohwi	12.04	*Spodiopogon sibiricus* Trin.	15.83
*Spodiopogon sibiricus* Trin.	8.47	*Pinus densiflora* Siebold & Zucc.	7.75	*Rhododendron mucronulatum* Turcz.	9.71
*Carex humilis* Leyss. var. *nana* (H.Lév. & Vaniot) Ohwi	8.05	*Rhododendron mucronulatum* Turcz.	7.05	*Carex humilis* Leyss. var. *nana* (H.Lév. & Vaniot) Ohwi	8.58
*Rhododendron mucronulatum* Turcz.	6.84	*Quercus mongolica* Fisch. ex Ledeb.	6.54	*Rhododendron schlippenbachii* Maxim.	7.93
*Lespedeza cyrtobotrya* Miq.	4.79	*Spodiopogon sibiricus* Trin.	6.13	*Lespedeza cyrtobotrya* Miq.	5.03
*Quercus mongolica* Fisch. ex Ledeb.	4.18	*Rhododendron schlippenbachii* Maxim.	5.62	*Calamagrostis arundinacea* (L.) Roth	3.91
*Quercus variabilis* Blume	3.94	*Calamagrostis arundinacea* (L.) Roth	4.28	*Atractylodes ovata* (Thunb.) DC.	3.10
*Atractylodes ovata* (Thunb.) DC.	3.58	*Atractylodes ovata* (Thunb.) DC.	3.63	*Lindera obtusiloba* Blume	3.10
*Calamagrostis arundinacea* (L.) Roth	3.21	*Lespedeza cyrtobotrya* Miq.	3.59	*Quercus mongolica* Fisch. ex Ledeb.	2.94

Group 1 consists of trees that died before 2008 or during the 2008–2011 period, representing a post-mortality stage of approximately 15 years. Group 2 includes trees that died between 2014 and 2017, with an elapsed time of approximately 7–10 years. Group 3 consists of trees that died between 2020 and 2021, representing a post-mortality stage of approximately 3 years.

### 3.3 Microbial community structure and diversity

**Figure 4:**
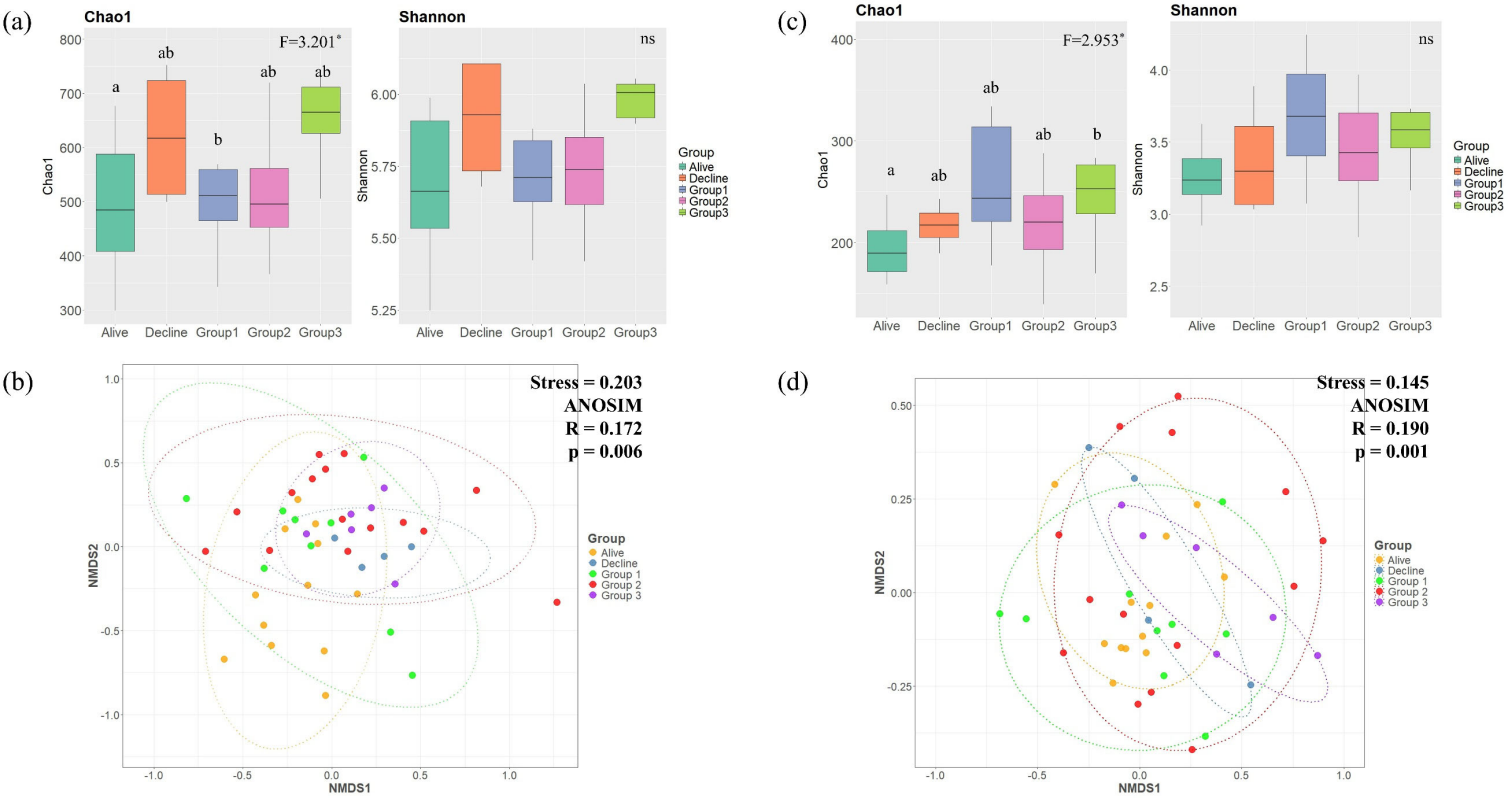
Comparison of α and β-diversity for fungal and bacterial communities associated with *P. densiflora* health status: healthy, declining, and dead (grouped by decay class). **(a)** Fungal α-diversity, assessed by Chao1 and Shannon indices. **(b)** Fungal β-diversity, visualized by non-metric multidimensional scaling (NMDS). **(c)** Bacterial α-diversity, assessed by Chao1 and Shannon indices. **(d)** Bacterial β-diversity, visualized by NMDS. Differences in α-diversity among groups were assessed using one-way ANOVA followed by a Bonferroni *post-hoc* test. For β-diversity, we used Non-metric Multidimensional Scaling (NMDS), and the significant differences among groups were tested using Analysis of Similarities (ANOSIM).

For fungi, in terms of diversity, healthy *P. densiflora* showed lower diversity than the other groups, and diversity was highest 15 or more years after tree death (**Figure 4a**). A statistically significant difference was observed only in the Chao1 index between the Alive and Decline groups, and no significant differences were found among the other groups (**Figure 4a**). In NMDS analysis, R was 0.172, indicating slight differences between groups, but mostly overlapping trends were observed (**Figure 4b**). In particular, the healthy *P. densiflora* group showed the most similar characteristics to Group 1, which had the longest duration since tree death, while Group 3 and the Decline group showed similar trends. Group 2, meanwhile, showed mixed characteristics. For bacteria, in terms of diversity, unlike fungi communities, diversity considerably increased in the Decline group and Group 3, followed by a gradual decrease (**Figure 4c**). Similar to the fungi community, a statistically significant difference was found only in the Chao1 index, and only between the Alive and Decline groups. In NMDS analysis, similar to fungi, we observed a low R-value (**Figure 4d**). However, the healthy *P. densiflora* group showed characteristics similar to those of Group 1, which had the longest duration since tree death. Some of Group 2 also showed similar characteristics to the healthy *P. densiflora* group, but overall showed a mixed distribution.

### 3.4 Microbial indicators of tree health, decline, and post-mortality

We performed analysis using an RF model to identify the main indicator genera driving differences between the groups, and the 15 taxa with the highest importance, alongside their relative abundance ratio, are shown in **Table 3**. The results showed that, for fungi, several genera in the Basidiomycota and Ascomycota phyla were the most important indicator taxa, whereas for bacteria, no taxa had notably high importance. Unclassified groups among Ascomycota (fungi) and Proteobacteria (bacteria) showed relatively high abundance in healthy *P. densiflora*, suggesting that these might play important roles in tree health. Meanwhile, *Oidiodendron* spp. and *Venturia* spp. in the phylum Ascomycota and *Umbelopsis* spp. in the phylum Mucoromycota showed high relative abundance in healthy *P. densiflora* and lower abundance in declining *P. densiflora*. This suggests that a decrease in these microbes could be related to the gradual decline of healthy *P. densiflora*. Fungi unclassified at the genus level, as well as *Russula* spp. and *Talaromyces* spp. in the phyla Basidiomycota and Ascomycota, and *Umbelopsis* spp. in the phylum Mucoromycota showed relatively high abundance in Group 2.

**Table 3:** Using the Random Forest model, we identified the relative abundance and importance (mean decrease in accuracy) of the top 15 taxa distinguishing tree health, decline, and post-mortality stages.

Domain	Phylum	Genus-level classification	Importance	Health	Decline	Group3	Group2	Group1
Fungi	Basidiomycota	Unclassified	0.124	0	0.31	0	0.677	0.013
Fungi	Ascomycota	Unclassified	0.096	0.011	0.288	0.047	0.562	0.092
Fungi	Ascomycota	Unclassified	0.093	0.583	0	0.353	0.064	0
Bacteria	Proteobacteria	Uncultured	0.089	0.662	0	0	0.203	0.135
Fungi	Basidiomycota	*Russula*	0.088	0.004	0	0.01	0.837	0.149
Fungi	Ascomycota	*Oidiodendron*	0.087	0.329	0.164	0.118	0.236	0.153
Fungi	Mucoromycota	*Umbelopsis*	0.086	0	0.018	0	0.839	0.143
Fungi	Ascomycota	*Venturia*	0.084	0.528	0.199	0.18	0.093	0
Fungi	Ascomycota	*Glutinomyces*	0.083	0.013	0.266	0.368	0.14	0.213
Fungi	Ascomycota	*Talaromyces*	0.081	0	0	0	0.911	0.089
Fungi	Basidiomycota	*Mucronella*	0.080	0	0.218	0.558	0.222	0.002
Fungi	Basidiomycota	Unidentified	0.078	0.155	0.084	0.205	0.328	0.228
Bacteria	Actinobacteriota	*Mycobacterium*	0.076	0.334	0.077	0.078	0.278	0.233
Fungi	Mucoromycota	*Umbelopsis*	0.071	0.405	0.152	0.044	0.328	0.071
Fungi	Ascomycota	*Sagenomella*	0.071	0.396	0.301	0.049	0.076	0.178

Group 1 consists of trees that died before 2008 or during the 2008–2011 period, representing a post-mortality stage of approximately 15 years. Group 2 includes trees that died between 2014 and 2017, with an elapsed time of approximately 7–10 years. Group 3 consists of trees that died between 2020 and 2021, representing a post-mortality stage of approximately 3 years. Unclassified = identified to higher rank only; Uncultured = environmental sequence without cultured reference (phylogenetic placement known).

### 3.5 Microbial community network variations

**Figure 5:**
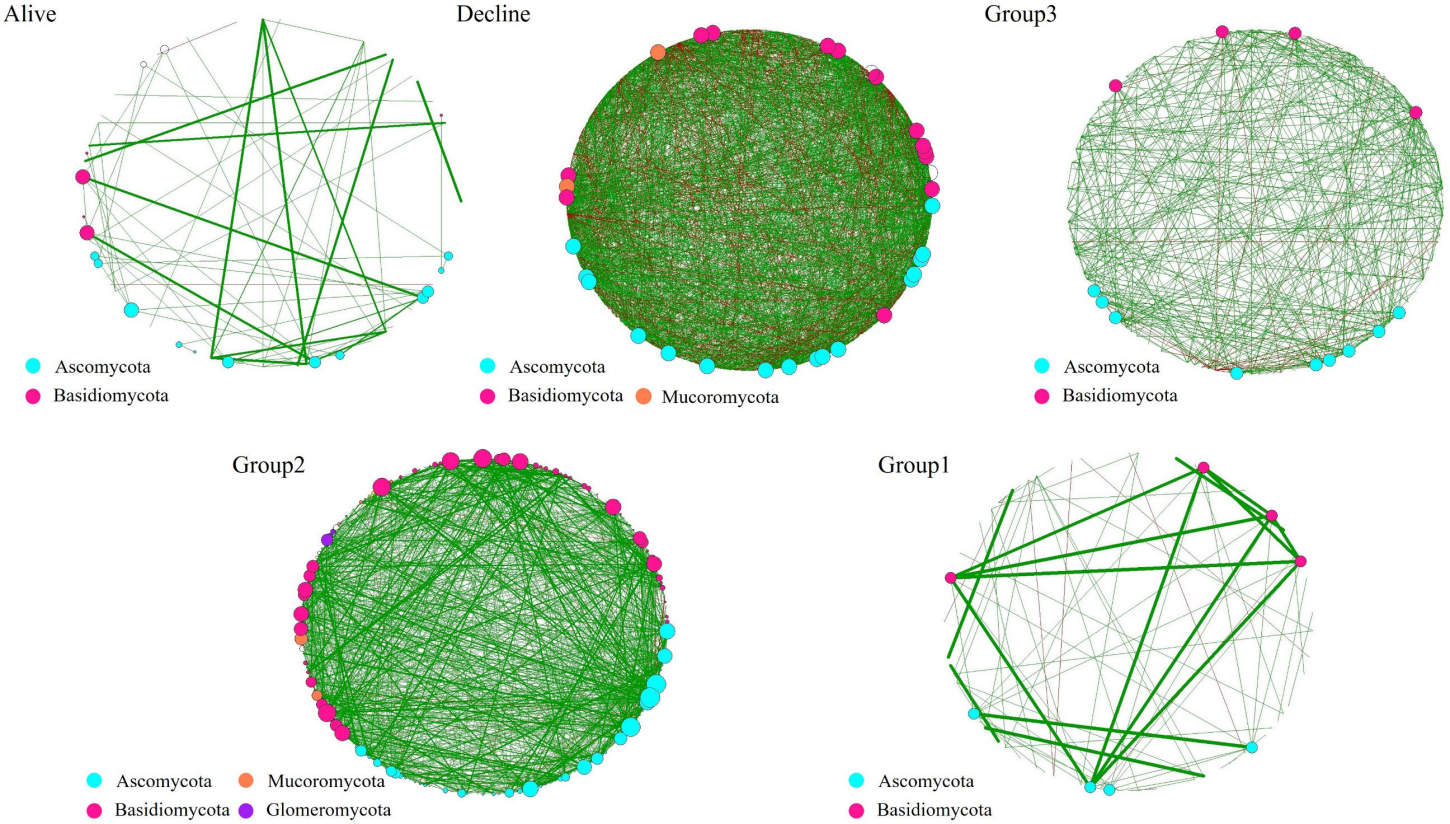
Co-occurrence networks of fungi according to the health status and mortality stages of *P. densiflora*. The analysis was conducted at the genus level and visualized at the phylum level. Genera with a frequency of 3 or less were categorized as “others” and depicted as black-outlined open circles. The size of each node is proportional to its degree, representing the number of connections with other nodes. Green and red links connect the nodes, representing positive and negative interactions, respectively.

We conducted network analyses separately for fungal and bacterial communities across five groups (Alive, Decline, and Mortality Groups 1–3). We primarily described the structural properties at the whole-network level, while using the Largest Connected Component (LCC) level for complementary analysis. In the co-occurrence network based on fungal groups, the main connectors at the phylum level were Mucoromycota, Basidiomycota, and Ascomycota, with Basidiomycota and Ascomycota making consistently high contributions to the network (**Figure 5**). We observed high modularity in healthy *P. densiflora* (whole network: 0.825, LCC: 0.563), indicating that specific microbial communities exhibited discernible network structures rather than random associations (**Table 4**). Meanwhile, in the Decline group, modularity was relatively high at the whole-network level (0.772) but dropped to –0.002 at the LCC level. Notably, this network contained a relatively large number of nodes and edges, and also exhibited a considerable proportion of negative correlations compared with the other groups. Group 3 showed a relative decrease in the number of nodes and edges compared to the Decline group, but exhibited modularity comparable to that of the Decline group (whole network: 0.849, LCC: –0.012). In Group 2, although the modularity of the whole-network level (0.407) was relatively low, the network at the LCC level (0.393) exhibited a modular organization where the main interactions were concentrated. In addition, the numbers of nodes and edges were relatively high at both the whole-network and LCC levels, forming a compact network structure. Meanwhile, in Group 1, modularity increased again at the whole-network level (0.802), whereas the modularity at the LCC level (–0.031) markedly decreased. In addition, the numbers of nodes and edges were similar to those in the Alive group.

**Table 4:** Network analysis metrics for fungal and bacterial communities associated with *P. densiflora*, categorized into healthy (Alive), decline, and dead groups (Group1–3).

Fungi										
Based on the whole-network level						Based on the largest connected component				
Classification	Ali	Dec	G3	G2	G1	Ali	Dec	G3	G2	G1
Number of nodes	136	195	162	215	120	19	33	13	157	8
Number of edges	63	1616	357	984	72	28	528	78	971	28
Modularity	0.825	0.772	0.849	0.407	0.802	0.563	–0.002	–0.012	0.393	–0.031
Average degree	0.93	16.57	4.41	9.15	1.20	2.95	32	12	12.37	7
Bacteria										
Number of nodes	209	225	182	231	177	122	29	5	87	30
Number of edges	722	1,457	51	242	147	694	406	10	203	94
Modularity	0.387	0.868	0.918	0.664	0.647	0.348	–0.002	–0.080	0.582	0.229
Average degree	6.91	12.95	0.56	2.10	1.66	11.38	28	4	4.67	6.27

Metrics include the number of nodes, number of edges, modularity, and average degree. Separate analyses were conducted for fungi and bacteria. Ali, alive *P. densiflora* stands (control); G1, Group 1; G2, Group 2; G3, Group 3; Dec, decline *P. densiflora* stands.

**Figure 6:**
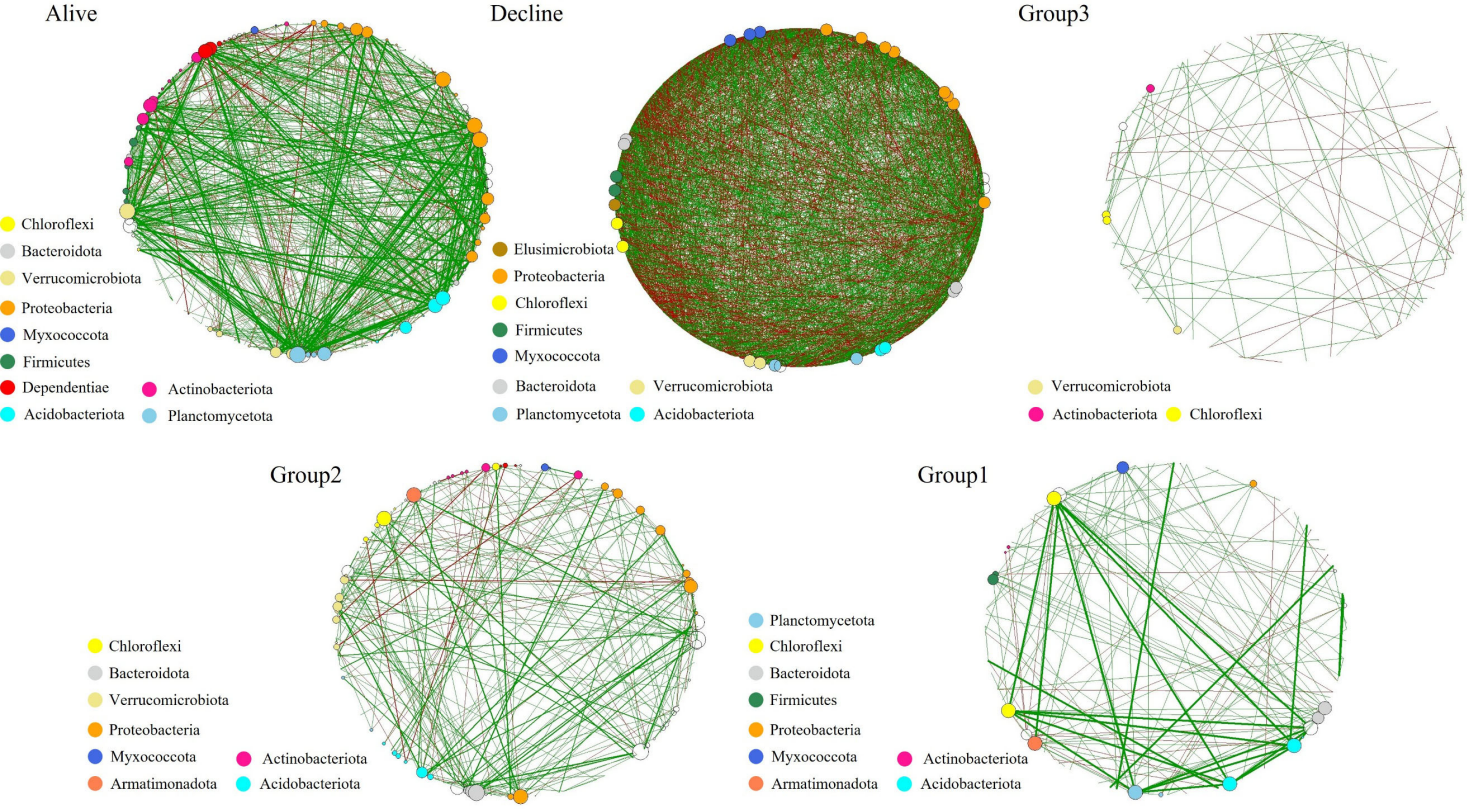
Co-occurrence networks of bacteria according to the health status and mortality stages of *P. densiflora*. The analysis was conducted at the genus level and visualized at the phylum level. Genera with a frequency of 3 or less were categorized as “others” and depicted as black-outlined open circles. The size of each node is proportional to its degree, representing the number of connections with other nodes. Green and red links connect the nodes, representing positive and negative interactions, respectively.

In terms of connectors at the phylum level, Proteobacteria, Verrucomicrobiota, Firmicutes, Chloroflexi, and Actinobacteriota showed consistently high contributions to the network across temporal changes in bacterial network structure (**Figure 6**). In healthy *P. densiflora* stands, modularity was relatively high compared with the other groups (whole network: 0.387; LCC: 0.348). At the whole-network level, the network comprised 209 nodes and 722 edges (**Table 4**). Meanwhile, in the Decline group, the number of edges (1,457) and nodes (225) markedly increased at the whole-network level, whereas they decreased at the LCC level (edges: 406, nodes: 29). Furthermore, modularity showed a marked increase at the whole-network level (0.868), while it dropped sharply at the LCC level (–0.002), and, similar to fungi, this group exhibited a relatively high proportion of negative correlations compared with the other groups. In Group 3, similar to the Decline group, modularity was high at the whole-network level (0.918) but low at the LCC level (–0.080). Meanwhile, the numbers of nodes and edges were the lowest among all groups. Consistent with the fungal network results, Group 2 showed lower modularity at the whole-network level (0.664) compared with the Decline and Group 3 networks, but markedly higher modularity at the LCC level (0.582). In addition, at the LCC level, Group 2 contained more nodes and edges than that of Group 3. In Group 1, modularity (whole network: 0.647, LCC: 0.229) was lower than in Group 2 but higher than in the Decline and Group 3 groups, and the whole network comprised 177 nodes and 147 edges.

## 4 Discussion

### 4.1 Vegetation succession patterns following *P. densiflora* mortality

We compared the time of multiple-tree mortality and CWD decay classes in *P. densiflora* forests, and found that after the death of *P. densiflora*, the CWD generally decayed from snag forms into logs and stumps, following a similar trajectory depending on the elapsed time since tree death. Although these findings align with predictable natural decay processes, it is significant that we quantitatively demonstrated this progression at specific time intervals, tracking the conversion from snags into logs and stumps through changes in decay classes. Indeed, our observations primarily reflect this common decay sequence. However, it is important to acknowledge that this observed decay progression does not comprehensively represent all forest dynamics, as live trees can also directly fall to logs due to windthrow or other disturbances, bypassing an intermediate snag stage. Consequently, our results should be interpreted with caution, recognizing inherent variability and complexity in forest stand dynamics. Meanwhile, this dataset is particularly valuable because it serves as a reference for estimating the timing of tree death across diverse regions based on dead-tree decay classes.

Historically, various forest gap sizes have been reported to alter light availability within stands, inducing natural disturbances in the area (Canham, 1988; Bolton and D’Amato, 2011; Lee et al., 2024). According to our findings, healthy *P. densiflora* forests and Group 3 and some Group 2 sites, which are considered to be at an early stage after tree death, showed similar species composition and did not show major differences. The species that appeared usually grow in the understory vegetation of pine forests on ridges. Meanwhile, Group 1, at least 15 years after tree death, showed relatively favorable levels of elements that affect soil richness (**Supplementary Table S5**). In addition, we observed the entry and regeneration of *P. densiflora* and *Quercus* spp. in the understory vegetation. One of the shared characteristics of these sites is that as dead *P. densiflora* decayed, forest gaps were opened. In other words, even though the light environment improved, the entry of woody plants other than *P. densiflora* was slow, and this is because *P. densiflora* habitats are usually on ridges (or upper slopes), where strong winds, ample sunlight, and a low topographic wetness index create low soil richness and harsh conditions. These environmental factors are important in allowing *P. densiflora* to be dominant as an edaphic climax, suggesting the potential for continual regeneration of this species. In particular, the fact that Quercus spp., which has relatively strong shade tolerance, was established as a succession species alongside *P. densiflora* is similar to findings in several previous studies in temperate regions (Higo et al., 1995; Beon and Bartsch, 2003; Choung et al., 2020). This supports the notion that this is a typical transition from *P. densiflora* forests to *P. densiflora*-broadleaved mixed forests. Furthermore, many previous studies have reported that the progressive increase in CWD decay in the stand over time gradually becomes an important factor affecting the soil environment (Harmon et al., 1986; Noh et al., 2017). Indeed, considering that Group 1 showed overall favorable levels of elements affecting soil richness compared to other groups (**Supplementary Table S5**), we cannot exclude the possibility that these changes affected the processes of plant entry and regeneration. However, there are limitations to discussing this clearly based on the current study, given that various ecological dynamic processes act in concert, including climate and local environmental characteristics (Sturtevant et al., 1997; Laiho and Prescott, 2004). Conducting further studies to better understand the processes of CWD decay, changes in the soil environment, and the relationships with species entry would contribute to even more detailed knowledge of the processes of change in aboveground stand behaviors in *P. densiflora* forests. To support practical forest management decisions, future research should specifically investigate the interactive effects of CWD decay stages and soil nutrient dynamics on species establishment success. Furthermore, in sub-alpine coniferous forests in South Korea, the entry of diverse species into forest gaps that appear as dominant tree species die reportedly causes rapid changes in forest structure (Lee et al., 2024). In lowland *P. densiflora* forests, even though multiple-tree mortality of *P. densiflora* leads to further expansion of forest gaps over time, we observed regeneration of *P. densiflora* and slow entry of other species. This shows differences in the pattern of species entry after the death of dominant tree species in high- and low-altitude coniferous forests. Furthermore, canopy openness was not identified as a significant variable distinguishing the multiple-tree mortality sites. This might be because the statistical difference did not emerge as the forest gap between Group1 and Group2 did not show a significant difference. However, as shown in **Figure 2**, it is suggested that the forest gap is gradually expanding as snags decay and fall over time. Furthermore, just as Group1 and some of Group2 showed a high association with rock exposure, it is possible that the formation of gaps and substrate characteristics (e.g., soil, moss cover, rock exposure) interacted to promote the regeneration of *P. densiflora* saplings. In fact, a study on *Picea abies* by Kathke and Bruelheide (2010) also found that gap age itself did not significantly affect regeneration density; however, a clear difference was confirmed in analyses that included microsites. Therefore, although canopy openness alone could not explain the differences between sites in this study, the observed variance in *P. densiflora* regeneration may be the result of an interaction effect between microsites (e.g., environments with high rock exposure) and gap size. To closely verify these effects, continuous and precise monitoring is required, and it is necessary to strengthen the interpretation and verification of dynamic changes in declining stands through comparative studies across various regions.

### 4.2 Temporal dynamics of microbial communities

We compared changes in the interactions and network structure between microbes and used an RF model to track differences in the composition of microbial communities between groups, thereby providing a theoretical basis for detecting the early signs of tree decline. In addition, we analyzed how microbial community composition is affected by changes in the degree of decay with time after tree death. This analysis addresses the limitations of aboveground observations and bridges the gap in existing knowledge regarding plant–microbe interactions and the resilience of forest ecosystems.

According to our results, healthy *P. densiflora* exhibited lower or similar bacterial and fungal diversity (Chao1 and H’) compared to declining or dead trees. This finding contrasts with studies on sub-alpine *Abies koreana* (Kang et al., 2024) and previous reports associating high diversity with enhanced pathogen suppression (Hu et al., 2016; Wagg et al., 2021). These discrepancies suggest that the relationship between microbial diversity and tree health is not universally linear but likely varies depending on host species and local environmental conditions. This observation supports the view that the abundance of specific functional groups is a more critical determinant of stress resistance than overall diversity (Hol et al., 2015; Kong et al., 2022). Crucially, our RF model results substantiate this perspective by identifying *Oidiodendron* spp. (Ascomycota) and *Umbelopsis* spp. (Mucoromycota) as key taxa significantly more abundant in healthy *P. densiflora*. *Oidiodendron* spp. are known ericoid mycorrhizal fungi that form symbiotic associations with plant roots and promote root growth (Perotto et al., 2012; Baba et al., 2021), while *Umbelopsis* spp. are reportedly effective at inhibiting phytopathogens near the roots and have positive effects on plant growth (Kolp et al., 2018; Wang et al., 2023). Therefore, forest managers should compare microbial communities between healthy and declining trees to identify taxa consistently associated with tree health. Monitoring these functionally important groups, rather than relying solely on overall diversity metrics, may provide clearer diagnostics of forest condition and more actionable guidance for management. Taken together, these rhizosphere microbial communities hold significant potential as early indicators for evaluating ecosystem resilience and informing conservation strategies (Mendes et al., 2013; Egidi et al., 2019).

In healthy *P. densiflora*, the rhizosphere microbial networks exhibited a relatively simple structure, characterized by fewer nodes and edges (particularly in fungi) but high modularity at both the whole-network and LCC levels. These results differ from a previous study on *Pinus sylvestris* growing in the southeast of Spain. Lasa et al. (2022) reported that the microbial networks of healthy *P. sylvestris* were more complex than those of declining individuals. In addition, several recent studies have proposed that compact networks with strong interactions are more favorable for maintaining plant health and suppressing pathogens than relatively simple networks (Tao et al., 2018; Yang et al., 2017; Fernández-González et al., 2020). Therefore, the simple network structure for healthy *P. densiflora* observed in our study contrasts previous studies and could be due to ecological differences, including tree species, local environment, or climatic conditions. Mendes et al. (2013) proposed that the microbial network structure of healthy plants is closely related to the concepts of core and minimal microbiomes. The core microbiome is hypothesized to maintain a minimal microbial community through functional duplication while effectively performing specific functions, such as maintaining plant health and suppressing pathogens (Mendes et al., 2011, 2013). Taken together, these findings suggest that, independent of the structural properties or forms of the network, *P. densiflora* regulates symbiotic relationships by selectively maintaining functionally important microbial groups (i.e., microbial communities associated with tree health), thus protecting itself from soil-mediated disease (Cook et al., 1995; Mendes et al., 2011, 2013). Moreover, after *P. densiflora* death, with the gradual opening of forest gaps, changes in stand productivity (Brumme, 1995; Prescott, 2002) and in the distribution of microclimatic conditions and substrate due to fallen trees (i.e., logs; Perreault et al., 2021) cannot be completely excluded. However, we did not observe statistically significant differences in the soil environment between the control and multiple-tree mortality sites (**Supplementary Table S10**). Even though large forest gaps were formed in the stands a long time after tree death (i.e., Group 1, over 15 years), microbial network complexity (particularly in fungal communities) resembled that of healthy *P. densiflora*. Thus, we did not find evidence to suggest that forest gaps and CWD had important effects on the rhizosphere soil environment.

Interestingly, compared with the Healthy group, networks in the Decline group were larger and more densely interconnected (increased nodes and edges) and showed a higher proportion of negative associations, while modularity decreased—particularly at the LCC level—indicating that the largest connected component became more intricately connected yet less compartmentalized. Similar to these characteristics, De Vries et al. (2018) showed that experimental drought made soil bacterial co-occurrence networks larger and more highly connected while reducing their modularity, and interpreted these changes as destabilising for the community. Theoretical work on microbial networks further suggests that community stability tends to be higher when interactions among community members are generally weak and the network is organized into modular, partially compartmentalized subcommunities (Coyte et al., 2015). In this context, the complex, densely connected yet weakly modular structure of the Decline network may be more closely linked to a transient, stress-related reorganization driven by competitive processes than to a stable equilibrium state. Hernandez et al. (2021) linked stress-induced reductions in network modularity and shifts in the balance between negative and positive associations to lower community stability, although in their case the relative contribution of negative associations decreased rather than increased. These similarities and contrasts suggest that such stress-related network responses may differ among host species and site conditions, highlighting the need for comparative studies across diverse tree species and regions.

Meanwhile, the microbial network in Group 2 exhibited a marked increase in both nodes and edges, characterized by high modularity. The changes in network structure could be due to exudates from mycorrhizal fungi increasing interactions between specific bacteria and fungi and altered community structure, as reported in a previous study (Hassani et al., 2018). As trees decline and die, organic matter is released through the degradation of wood by root mycorrhizal fungi (Ekblad et al., 2013). This released organic matter could promote various microbial interactions, resulting in a rapid increase in population sizes and the formation of complex networks (Ehleringer et al., 2000; Clemmensen et al., 2015). While our RF analysis indicated that certain fungal genera were relatively enriched during this stage, these findings should be viewed as exploratory associations rather than causal evidence, given the limitations of genus-level identification. Importantly, this elevated complexity was transient: by approximately 15 years after mortality, the networks reverted to a simple structure similar to that of healthy *P. densiflora*, with reduced numbers of nodes and edges, and whole-network level modularity tended to return to similar levels. These patterns appear to reflect a short-term reorganization of belowground interactions following mortality, possibly driven by shifts in resource availability and the transient expansion of specific microbial groups, with a subsequent trend toward community re-balancing over time. We therefore view changes in network connectivity as community-scale indicators of belowground dynamics rather than evidence for taxon-specific mechanisms. From a management perspective, tracking these temporal shifts in network complexity could offer a practical, complementary signal for assessing forest condition under climate-driven disturbance. However, to establish this approach as a diagnostic tool, future research is essential. Integrative, functionally resolved longitudinal studies are needed to establish causal links between network structure and ecosystem processes and to clarify the roles of taxa active during forest dynamics.

### 4.3 Linkages between above- and belowground ecosystems: insights and limitations

In our study, we found that, after the multiple-tree mortality of *P. densiflora*, both aboveground species composition and rhizosphere microbial network structure—particularly fungal communities—gradually tended to return toward conditions similar to those in healthy *P. densiflora* forests. Despite improved light availability as dead trees decayed and forest gaps expanded, the establishment of other tree species remained limited, and regeneration was consistently dominated by *P. densiflora*. In this chapter, we hypothesize that this regeneration process may have been driven by intimate interactions between the above- and belowground environments. Specifically, we propose that microbial communities, including mycorrhizal fungi, could have contributed to the re-establishment of *P. densiflora*, with resource sharing via Common Mycorrhizal Networks (CMNs) serving as one possible mechanism. In our chronosequence, RF analysis revealed that the mid-decay period (~7–10 years after death) was characterized by an increased representation of genera such as *Talaromyces* and *Russula*. *Talaromyces* spp. have been reported to include antifungal and plant-growth–promoting strains, whereas many *Russula* spp. form ectomycorrhizae that have been associated with improved host nutrient and water acquisition and increased stress tolerance (Bergemann et al., 2006; Dickie and Moyersoen, 2008; Wang et al., 2015; Filizola et al., 2019). Snags can act as reservoirs for these fungi and, together with the rhizosphere fungi of living trees, may form shared mycorrhizal networks that facilitate resource sharing between dead and living *P. densiflora* (Toju et al., 2013; Figueiredo et al., 2021). By ≥15 years after death (Group 1), fungal co-occurrence networks tended to resemble those in healthy *P. densiflora* stands at the whole-network level, exhibiting comparable complexity and modularity. We speculate that this convergence might stem from non-exclusive processes, such as a potential decrease in exudate-responsive taxa or the development of functional CMN links between snags and living trees. These patterns suggest that interactions between above- and belowground components may be linked to tree regeneration dynamics and might evolve as the decay of *P. densiflora* progresses. However, direct, site-specific evidence demonstrating CMN connections is required. Because our identifications are limited to the genus level, and some assignments are incomplete, we cannot assign functional roles at our sites. The current results alone are clearly limited in supporting this hypothesis and will need to be augmented by empirical research that can demonstrate the necessary conditions for the belowground connectivity between plants via the CMN (Figueriedo et al., 2021).

In addition, specific evidence is needed to confirm whether resources are transferred between plants directly via the CMN (Bever et al., 2010; Courty et al., 2010). Moreover, CMN formation does not guarantee that the effects of these interactions will always benefit plants, as the outcomes may include benefits and disadvantages depending on the circumstances, requiring further investigations (Bücking et al., 2016; Figueriedo et al., 2021). If we can develop a clearer understanding of these interactions through future research, it could help us to understand ecological resilience and processes of regeneration in coniferous forests that are declining due to environmental stress.

## 5 Conclusion

We analyzed temporal changes in above- and belowground stand behaviors following multiple-tree mortality of *P. densiflora*, thereby contributing to a deeper understanding of ecological change patterns and the recovery processes of this species. Forest gaps formed by the multiple-tree mortality of *P. densiflora* changed depending on the time of tree death and increased light availability in the stand but did not lead to major changes in the species composition of understory vegetation. Moreover, when 15 or more years had passed since tree death, we observed active *P. densiflora* regeneration. Besides ample sunlight, major *P. densiflora* habitats have harsh environmental conditions, such as strong winds, which appear to favor the persistent dominance of *P. densiflora* as an edaphic climax. Meanwhile, the composition of belowground microbial communities showed subtle shifts along the decline and decay-class gradient and weak but detectable differences from healthy *P. densiflora*, while overall exhibiting largely overlapping features. Moreover, RF model and α-diversity analyses demonstrated that the presence and abundance of specific functional groups in *P. densiflora* could be an important indicator for evaluating tree health. Network analysis of microbial communities revealed structural shifts depending on tree health and decay class. In particular, fungal co-occurrence networks at the whole-network level in snags 15 years or more after tree death (decay class 6) exhibited a structure similar to that of healthy *P. densiflora*. This study tracks continuous changes within the forest ecosystem under changing environmental conditions, thereby deepening our understanding of stand-level ecological dynamics and providing insights into improving the adaptive capacity of sustainable coniferous forests. Furthermore, we hypothesize that the close interaction between above- and belowground components may be a key factor driving changes in these stands.

## Data Availability

The DNA sequencing data generated in this study have been deposited in the NCBI BioProject database under accession numbers PRJNA1372408, PRJNA1372409. Additional data supporting the findings of this study are available from the corresponding author and the first author upon reasonable request.
