# Supplementary material for: Above- and belowground succession following multiple-tree mortality in Pinus densiflora forests
Source: Front Plant Sci. 2026 Jan 22;16:1737673. doi: 10.3389/fpls.2025.1737673 (PMC12872792; doi:10.3389/fpls.2025.1737673)
Supplement: Supplementary file 1 [file DataSheet1.docx]

Supplementary Material

Above- and belowground succession following multiple-tree mortality in *Pinus densiflora* forests

Seung–Jae Lee^a^, Ah–Rim Lee^a^, Dong–Seok Son^b^, Dong–Bin Shin^a^, Seok Hui Lee^a^, Yeong-Eun Kim^a^, Jun Won Kang^c^, Seung–Hwan Oh^c,*^

^a^Department of Forestry, The Graduate School of Kyungpook National University, Daegu 41566, Republic of Korea
^b^Wetland Center, National Institute of Ecology, Changnyeong 50303, Republic of Korea
^c^School of Forest Sciences and Landscape Architecture, Kyungpook National University, Daegu 41566, Republic of Korea

*** Correspondence:**Seung-Hwan Oh (oshwan@knu.ac.kr)

Keywords: *Pinus densiflora*_1_, Microbial communities_2_, Climate change_3_, Vegetation succession_4_, Rhizosphere soil_5_, Coarse woody debris_6_

# Supplementary Figures

**Figure S1.** Status of *P. densiflora* Multiple-Tree Mortality Sites. (a) shows an example of identifying a multiple-tree mortality area using orthographic imagery produced with drone footage. (b) represents a site where tree mortality occurred between 2020 and 2021, classified as Group 3. As of 2024, approximately three years have passed since tree death, making it the most recent multiple-tree mortality site. Small branches and pinecones remain on the dead trees. (c) corresponds to Group 2, where mortality occurred between 2013 and 2017. As of 2024, this site represents an intermediate stage of multiple-tree mortality, with 7–10 years having passed since tree death. Thick branches are still present. (d) and (e) are classified as Group 1, where tree mortality occurred before 2008 or between 2008 and 2011. As of 2024, these are the oldest multiple-tree mortality sites, with more than 15 years having passed since tree death. Regeneration of *P. densiflora* saplings and the transformation of snags into log forms can be observed.

**Figure S2.** The comparison of meteorological data between the past (1972–1990) and the recent approximately 30 years (1990–2023) using data from the Uljin-gun meteorological administration: (a) shows the results based on data from 1972 to 1990, and (b) shows the results based on data from 1990 to 2023.

**Figure S3.** The snag and log decay class were determined based on the stand-level biodiversity monitoring protocol steps for field data collection from British Columbia (Province of British Columbia, 2009).

**Figure S4.** This figure presents the results of historical aerial photograph analysis used to determine the timing of tree death at *P. densiflora* multiple-tree mortality sites. As examples, historical aerial images from two out of the 15 total sites are shown. Among the year labels in the images, the years highlighted in bold red text indicate the point at which tree mortality was first observed; this year was defined as the timing of tree death for the corresponding multiple-tree mortality site.

**Figure S5.** Summary of the analysis of tree death timing at 15 *P. densiflora* multiple-tree mortality sites using historical aerial photographs. The timing of tree death was broadly categorized into three groups. Group 1 comprises six samples, Group 2 seven samples, and Group 3 two samples. When only a single symbol is shown for a given year, we inferred that tree mortality occurred in that year. An exception applies to years prior to 2008: because earlier aerial photographs are not available, plots identified as mortality sites in the 2008 imagery were interpreted as having died sometime before 2008. When the estimated timing of mortality extended across consecutive years, we represented this as a line segment connecting those years. For example, in site 14, trees appeared alive in the 2008 aerial photographs but were confirmed as dead in the 2011 imagery; thus, we inferred that mortality occurred between 2009 and 2011 and indicated this interval with a connecting line. For definitions of each group, please refer to the main text or the caption of Fig. S1.

**Figure S6.** Field photograph of the Decline group during the microbial community analysis of *Pinus densiflora*. Defoliation was assessed based on the method of Huo et al. (2019), and the results indicated a defoliation range of 40–80%.

Figure S7. Random forest hyperparameter testing status for identifying and evaluating the importance of indicator taxa that determine bacterial and fungal community differences among the four groups (Group1–3 & Decline). The evaluation was conducted using Out-of-Bag (OOB) with mtry and ntree. Refer to the main text for the definitions of Group1–3 and Decline groups.

**
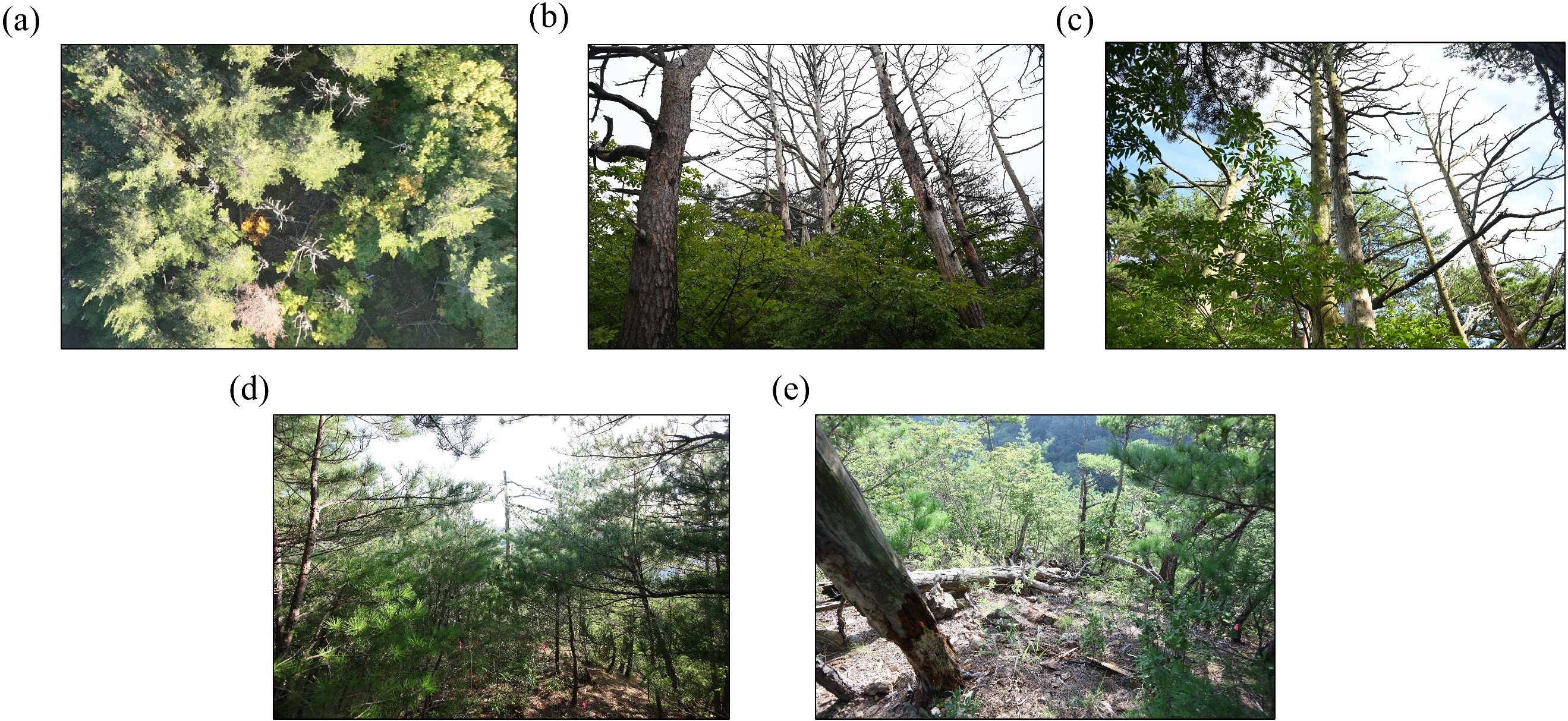
**

**Figure S1.** Status of *P. densiflora* Multiple-Tree Mortality Sites. (a) shows an example of identifying a multiple-tree mortality area using orthographic imagery produced with drone footage. (b) represents a site where tree mortality occurred between 2020 and 2021, classified as Group 3. As of 2024, approximately three years have passed since tree death, making it the most recent multiple-tree mortality site. Small branches and pinecones remain on the dead trees. (c) corresponds to Group 2, where mortality occurred between 2013 and 2017. As of 2024, this site represents an intermediate stage of multiple-tree mortality, with 7–10 years having passed since tree death. Thick branches are still present. (d) and (e) are classified as Group 1, where tree mortality occurred before 2008 or between 2008 and 2011. As of 2024, these are the oldest multiple-tree mortality sites, with more than 15 years having passed since tree death. Regeneration of *P. densiflora* saplings and the transformation of snags into log forms can be observed.


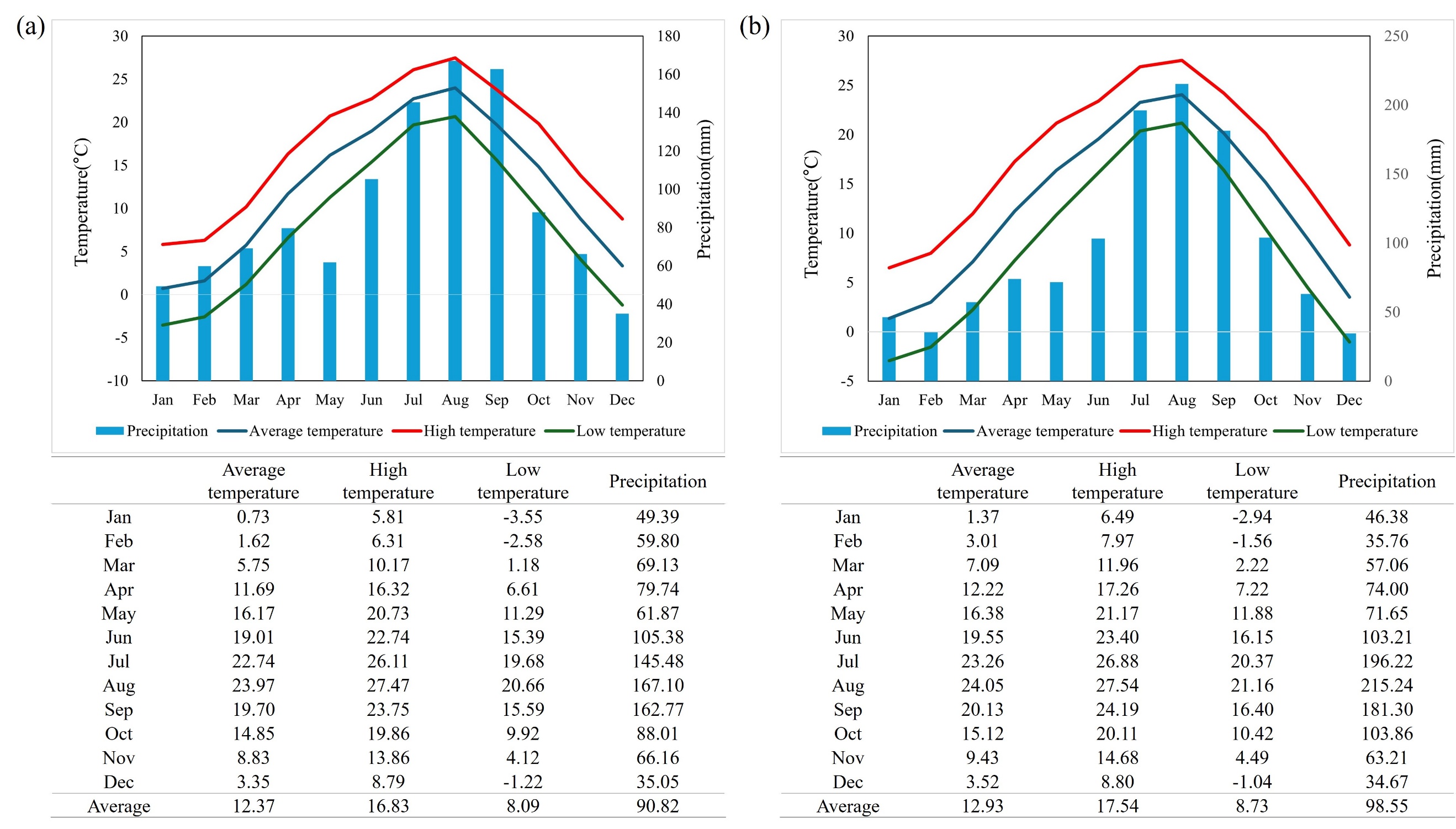


Figure S2. The comparison of meteorological data between the past (1972–1990) and the recent approximately 30 years (1990–2023) using data from the Uljin- gun meteorological administration: (a) shows the results based on data from 1972 to 1990, and (b) shows the results based on data from 1990 to 2023.


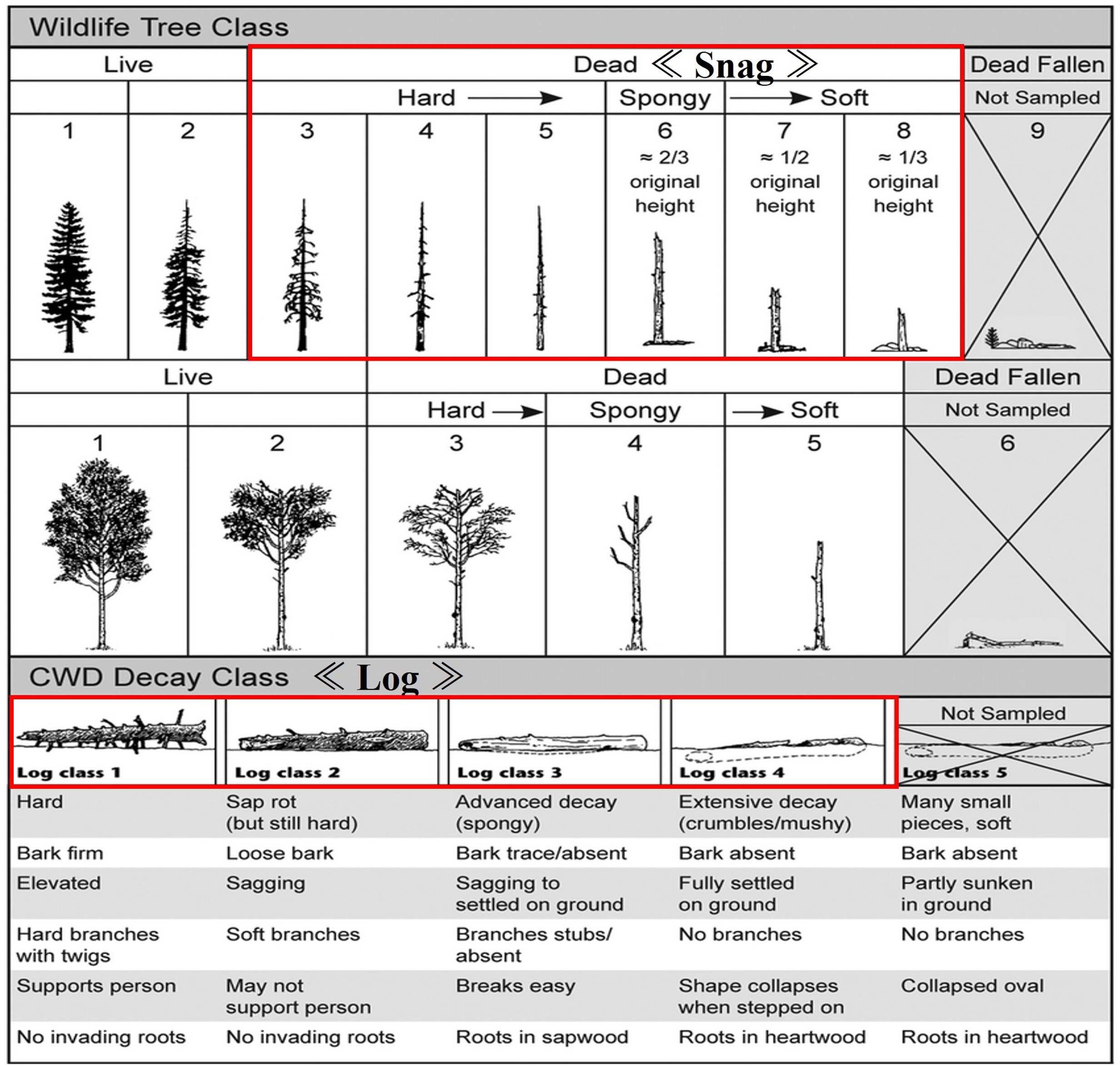


Figure S3. The snag and log decay class were determined based on the stand-level biodiversity monitoring protocol steps for field data collection from British Columbia (Province of British Columbia, 2009). We assessed the decay class with reference to the area marked by the red box.


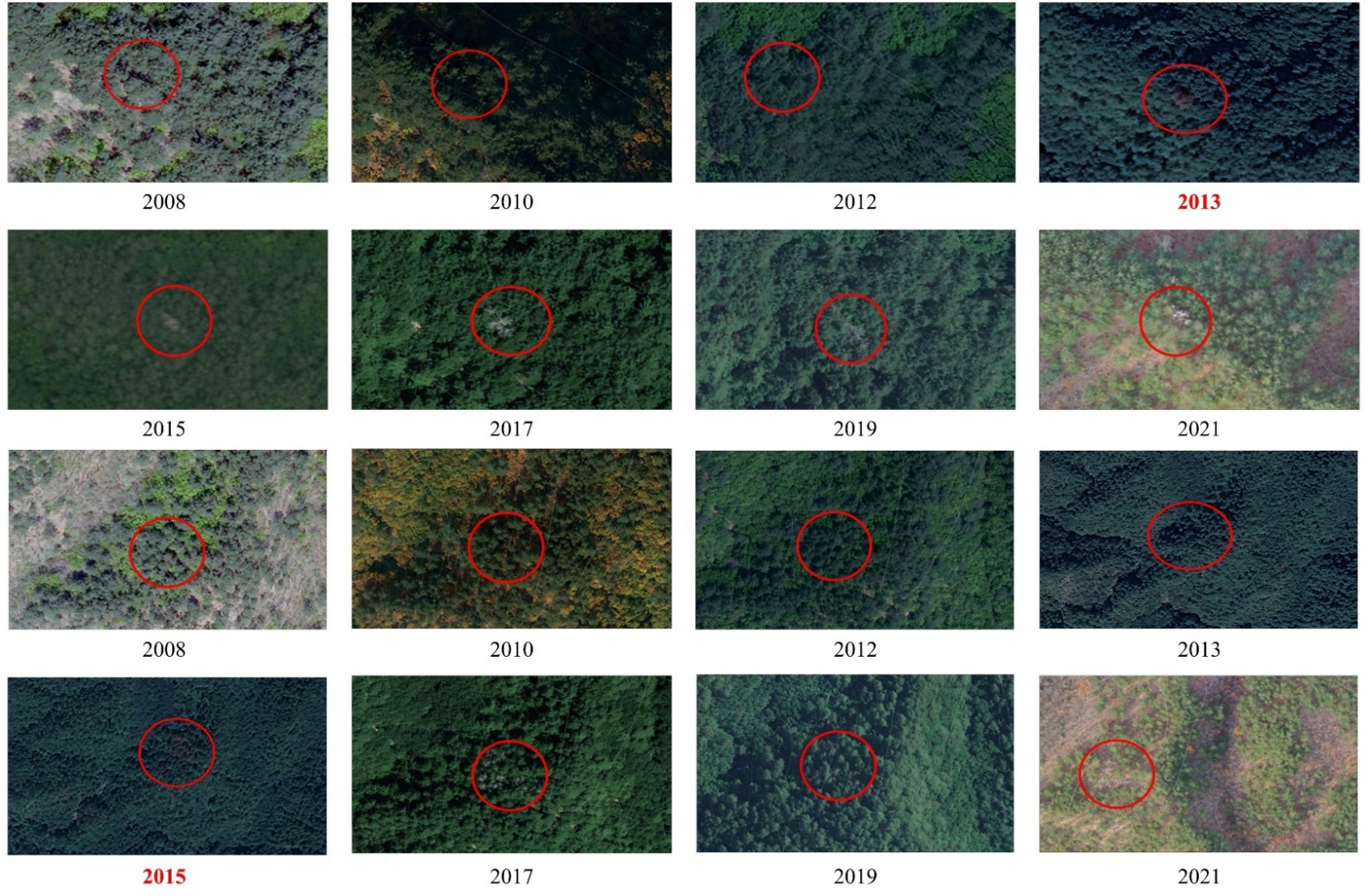


Figure S4. This figure presents the results of historical aerial photograph analysis used to determine the timing of tree death at *P. densiflora* multiple-tree mortality sites. As examples, historical aerial images from two out of the 15 total sites are shown. Among the year labels in the images, the years highlighted in bold red text indicate the point at which tree mortality was first observed; this year was defined as the timing of tree death for the corresponding multiple-tree mortality site.


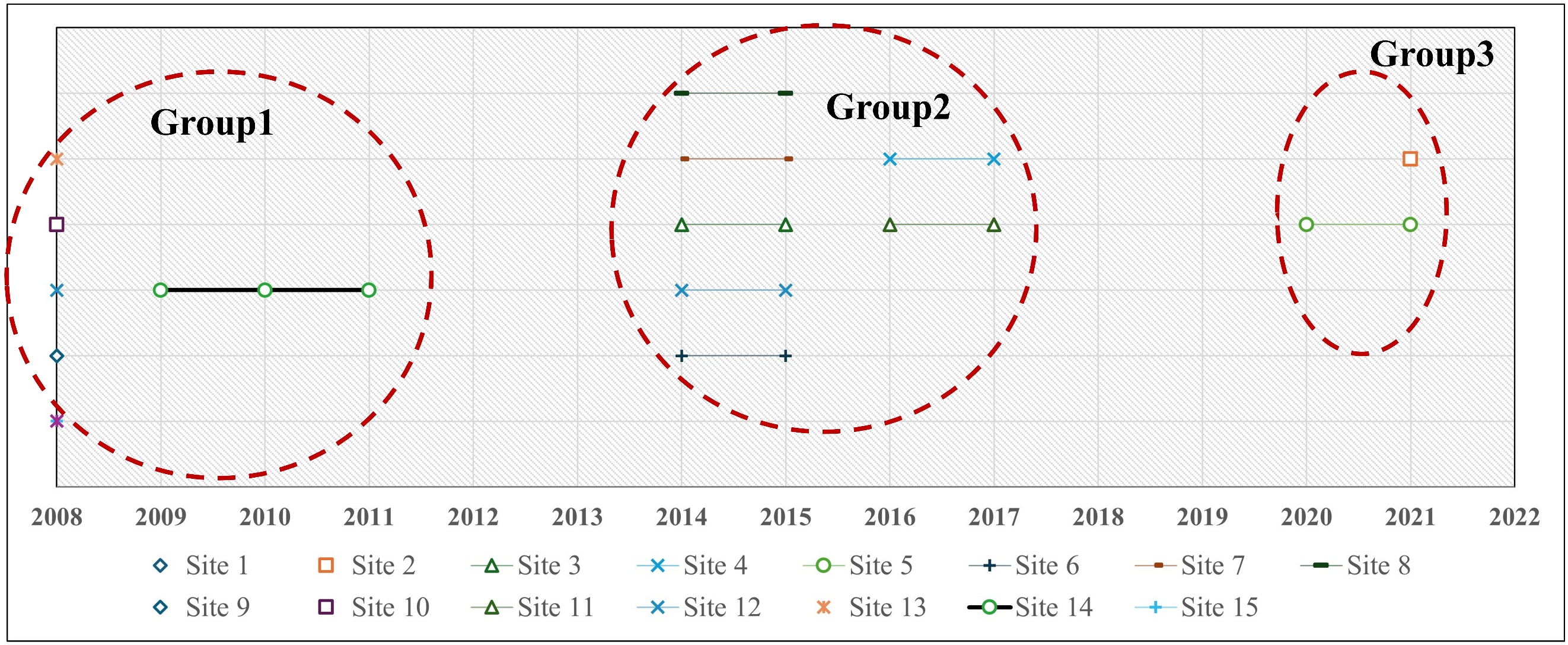


Figure S5. Summary of the analysis of tree death timing at 15 *P. densiflora* multiple-tree mortality sites using historical aerial photographs. The timing of tree death was broadly categorized into three groups. Group 1 comprises six samples, Group 2 seven samples, and Group 3 two samples. When only a single symbol is shown for a given year, we inferred that tree mortality occurred in that year. An exception applies to years prior to 2008: because earlier aerial photographs are not available, plots identified as mortality sites in the 2008 imagery were interpreted as having died sometime before 2008. When the estimated timing of mortality extended across consecutive years, we represented this as a line segment connecting those years. For example, in site 14, trees appeared alive in the 2008 aerial photographs but were confirmed as dead in the 2011 imagery; thus, we inferred that mortality occurred between 2009 and 2011 and indicated this interval with a connecting line. For definitions of each group, please refer to the main text or the caption of Fig. S1.


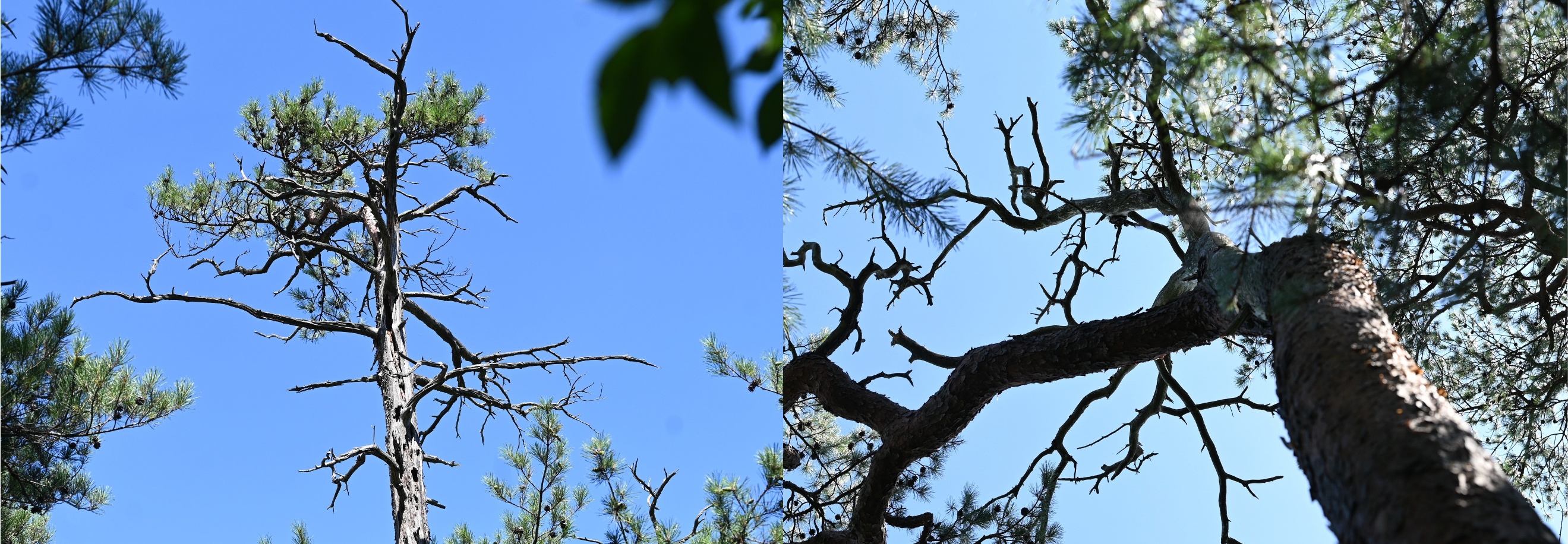


Figure S6. Field photograph of the Decline group during the microbial community analysis of *Pinus densiflora*. Defoliation was assessed based on the method of Huo et al. (2019), and the results indicated a defoliation range of 40–80%.


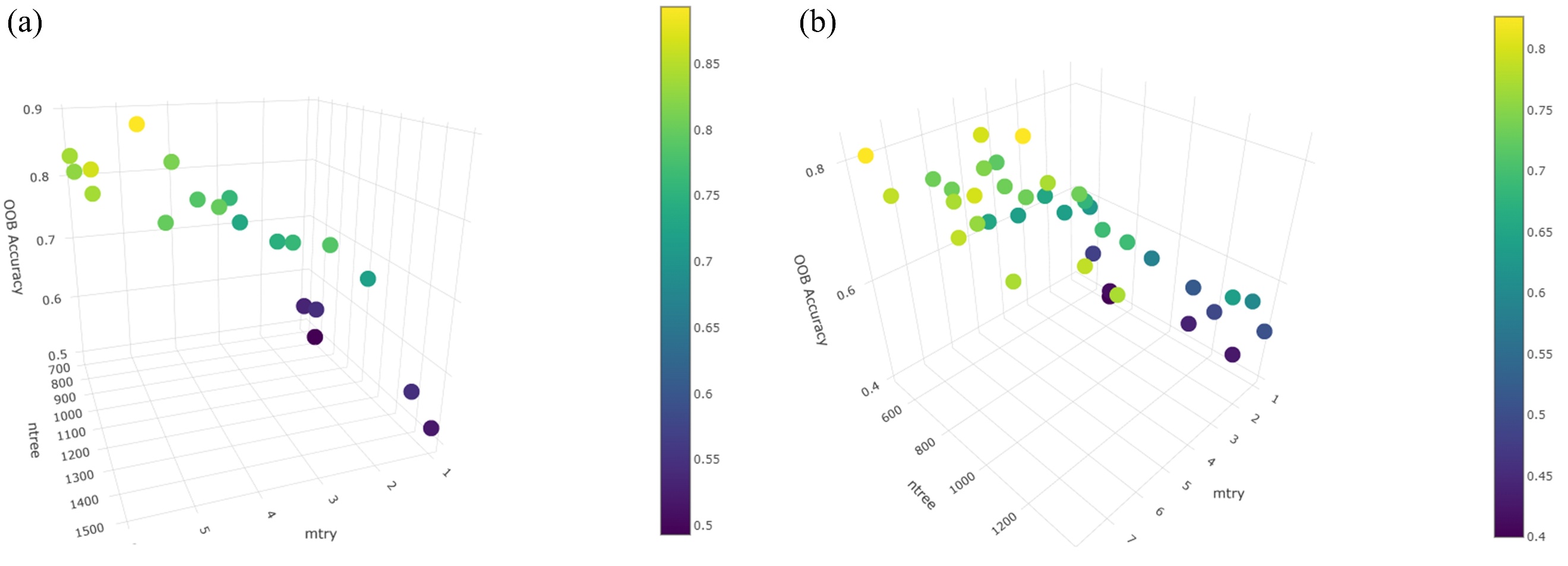


Figure S7. Random forest hyperparameter testing status for identifying and evaluating the importance of indicator taxa that determine bacterial and fungal community differences among the four groups (Group1–3 & Decline). The evaluation was conducted using Out-of-Bag (OOB) with mtry and ntree. Refer to the main text for the definitions of Group1–3 and Decline groups.

# Supplementary Tables

**Table S1.** The area status by forest type within the Wangpicheon Ecosystem and Landscape Conservation Area (ELCA). The forest types were classified based on the Korea Forest Service (KFS) Forest Type Map.

**Table S2.** Decay Characteristics of Stumps by Stage. Modified classification of stump decay classes based on the study by Rouvinen & Kouki (2002), incorporating field survey results.

**Table S3.** Topographic and environmental characteristics and distribution of coarse woody debris at 15 multiple-tree mortality sites.

**Table S4.** Explanation of soil chemical properties and sampling methods for each sample.

**Table S5.** The table shows the mean values of soil chemical properties across five groups, with the numbers in parentheses representing the standard deviation. The Control group refers to a healthy *P. densiflora* stand, while the definitions of the other groups are provided in the main text. The abbreviations are as follows: total nitrogen (N), exchangeable cations (K⁺, Ca²⁺, Mg²⁺, and Na⁺), available phosphorus (measured as Olsen-P), organic carbon (C), organic matter (OM), cation exchange capacity (CEC), soil pH, and electrical conductivity (EC).

**Table S6.** Primer sequences used for bacterial 16S rRNA (V4 region) and fungal ITS2 amplicon sequencing

**Table S7.** Frequency information for each processing step in the bacterial 16S rRNA V4 and fungal ITS2.

**Table S8.** Summary of statistical tests assessing the effect of plot area on species richness. Results are shown for a simple linear regression and a one-way analysis of variance (ANOVA) using plot area (m²) or plot-area class as predictors. Degrees of freedom (df; numerator, denominator) and the coefficient of determination (R²) are reported. Plot-area classes: 200, 225, 300, and 400 m². Including the control plots, there were 27 plots with an area of 200 m², and one plot each with areas of 225, 300, and 400 m².

**Table S9.** Random forest hyperparameter testing status for identifying and evaluating the importance of indicator taxa that determine bacterial and fungal community differences among the four groups (Group1–3 & Decline). The evaluation was conducted using Out-of-Bag (OOB) with mtry and ntree. Refer to the main text for the definitions of Group1–3 and Decline groups.

**Table S10.** Results of Paired sample t-Test on soil chemical properties between multiple-tree mortality sites and healthy *P. densiflora* forests (Control). No statistically significant differences were observed in soil chemical properties between the two groups. SD indicates standard deviation.

Table S1. The area status by forest type within the Wangpicheon Ecosystem and Landscape Conservation Area (ELCA). The forest types were classified based on the Korea Forest Service (KFS) Forest Type Map.

| Species | Area (㎢) | Ratio (%) |
| --- | --- | --- |
| *Pinus densiflora* Siebold & Zucc. | 35.37 | 34.40 |
| Other *Quercus* species | 23.35 | 22.70 |
| *Quercus mongolica* Fisch. ex Ledeb. | 13.91 | 13.52 |
| Other broadleaf species | 7.82 | 7.60 |
| Mixed coniferous and broadleaf forest | 7.22 | 7.02 |
| the others | 6.47 | 6.29 |
| *Quercus variabilis* Blume | 5.95 | 5.79 |
| *Pinus koraiensis* Siebold & Zucc. | 1.59 | 1.55 |
| *Larix kaempferi* (Lamb.) Carrière | 0.86 | 0.83 |
| *Pinus rigida* Mill. | 0.20 | 0.20 |
| *Betula pendula* Roth | 0.07 | 0.07 |
| *Castanea crenata* Siebold & Zucc. | 0.03 | 0.03 |
| *Alnus japonica* (Thunb.) Steud. | 0.01 | 0.00 |
| *Populus* × *canadensis* Moench | 0.00 | 0.00 |
| Sum | 102.84 | 100.0 |

**Table S2**. Decay Characteristics of Stumps by Stage. Modified classification of stump decay classes based on the study by Rouvinen & Kouki (2002), incorporating field survey results.

| Stump (1–3) | |
| --- | --- |
| Decay class | Description |
| 1 | Wood hard: a pushed knife penetrates only a few millimetres into the wood. |
| 2 | Wood is fairly hard: the knife penetrates approximately 1–2 cm into the wood. The bark is partially damaged, and the inner part has started to decay. |
| 3 | Wood is soft: the whole blade of the knife easily penetrates into the wood. The bark is damaged and consists of grid-like fragments. |
| 4 | Wood is very soft: almost completely decomposed and disintegrates easily between the fingers. The bark is completely peeled off, and the wood texture has turned into a soft powdery form. |

**Table S3**. Topographic and environmental characteristics and distribution of coarse woody debris at 15 multiple-tree mortality sites.

|  | Average | | Max | | Min | | |
| --- | --- | --- | --- | --- | --- | --- | --- |
| Elevation (m) | 564 | | 842 | | 414 | | |
| Slope (°) | 29 | | 40 | | 15 | | |
| Rock exposure | 16 | | 70 | | 5 | | |
|  | Ridge | | | Upper slope | | | |
| Topographical position | 7 | | | 8 | | | |
|  | North | West | | East | | | South |
| Aspect direction | 5 | 2 | | 1 | | | 7 |
| Current status of the number of CWDs within multiple-tree mortality sites | | | | | | | |
|  | Average | | Max | | Min | | |
| Snag | 9.5 | | 19 | | | 4 | |
| Log | 6 | | 15 | | | 0 | |
| Stump | 1.3 | | 5 | | | 0 | |

**Table S4**. Explanation of soil chemical properties and sampling methods for each sample.

| Division | Analytical method |
| --- | --- |
| total nitrogen (N) | Total nitrogen was determined by the Kjeldahl method, in which soil samples are digested with concentrated sulfuric acid and a catalyst, followed by distillation and titration to quantify nitrogen content. |
| available phosphorus (measured as Olsen-P) | Available phosphorus was determined using the Olsen method (0.5 M NaHCO₃, pH 8.5), which desorbs plant-available phosphate from soil colloids under mildly alkaline conditions. |
| organic matter (OM) | Organic carbon (OC) was determined using the Walkley–Black method. In this method, soil organic carbon is oxidized by K₂Cr₂O₇ in the presence of concentrated sulfuric acid, and the excess dichromate is back-titrated with standardized ferrous salt (e.g., FeSO₄·7H₂O or ferrous ammonium sulfate) using a ferroin (1,10-phenanthroline–ferrous) indicator. For the analysis, 0.5 g of air-dried, ground soil was placed in a 250 mL Erlenmeyer flask and treated with 10 mL of 1 N K₂Cr₂O₇, followed by the addition of 20–25 mL concentrated H₂SO₄; the mixture was gently swirled and allowed to stand for ~30 min before dilution (~200 mL) and back-titration. Organic matter (OM) was then estimated as 1.724 times OC (Van Bemmelen factor) (Page et al., 1982). |
| cation exchange capacity (CEC) | Cation exchange capacity (CEC) was determined by saturating soil exchange sites with 1 M ammonium acetate (NH₄OAc, pH 7.0). Excess electrolyte was removed by rinsing with 95% ethanol. The adsorbed NH₄⁺ was then displaced with 1 M KCl, and NH₄–N in the leachate was quantified by Kjeldahl distillation–titration. CEC was expressed as cmolc kg⁻¹. |
| soil pH | Soil pH was determined using the glass electrode method with a 1:5 (soil:water) suspension. For this, 5 g of air-dried soil was placed in a 50 mL beaker, mixed with 25 mL of distilled water, stirred with a glass rod, and left to stand for 1 hour before measurement with a pH meter. |
| EC  (Electrical conductivity) | Soil electrical conductivity was measured as EC1:5(H₂O, 25 °C) using a glass conductivity probe. Air-dried, sieved (<2 mm) soil was mixed with deionized water at a 1:5 (w/v) ratio, shaken for 30 min, allowed to settle for 30 min, and the supernatant was read at 25 °C with an ATC-equipped meter calibrated against 0.01 M KCl (1.413 dS m⁻¹). Results are expressed in dS m⁻¹. |
| exchangeable cations  (K⁺, Ca²⁺, Mg²⁺, and Na⁺) | Exchangeable cations (Ca²⁺, Mg²⁺, K⁺, and Na⁺) were extracted with 1 M ammonium acetate (NH₄OAc, pH 7.0) at a 1:10 soil:solution ratio from air-dried, < 2 mm-sieved soils, shaken for 30 min, and filtered. Ca+Mg were determined by EDTA titration at pH 10 using Eriochrome Black T; Ca was determined by EDTA titration at pH 12–13 2using murexide, and Mg was obtained by difference (Ca+Mg − Ca). K⁺ and Na⁺ in the same extract were quantified by flame photometry. Results were expressed as cmolc kg⁻¹. |

**Table S5**. The table shows the mean values of soil chemical properties acrossfive groups, with the numbers in parentheses representing the standard deviation. The Control group refers to a healthy *P. densiflora* stand, while the definitions of the other groups are provided in the main text. The abbreviations are as follows: total nitrogen (N), exchangeable cations (K⁺, Ca²⁺, Mg²⁺, and Na⁺), available phosphorus (measured as Olsen-P), organic carbon (C), organic matter (OM), cation exchange capacity (CEC), soil pH, and electrical c2onductivity (EC).

|  | Group1 | Group2 | Group3 | Decline | Control |
| --- | --- | --- | --- | --- | --- |
| Elevation (m) | 545(0.07) | 582(0.24) | 559(0.06) | 630 | 576(0.18) |
| Slope (°) | 29(0.23) | 31 (0.21) | 24(0.25) | 35 | 24(0.27) |
| pH [1:5] | 4.19(0.11) | 4.27(0.10) | 4.46(0.08) | 4.73 | 4.25(0.10) |
| EC (dS/m) | 0.06(0.39) | 0.09(0.50) | 0.08(0.63) | 0.04 | 0.08(0.53) |
| OM (%) | 6.22(0.40) | 5.29(0.41) | 5.88(0.02) | 3.90 | 5.55(0.36) |
| P_2_O_5_ (mg/kg) | 22.28(0.21) | 20.33(0.24) | 14.22(0.36) | 16.36 | 20.06(0.28) |
| N (%) | 0.15(0.50) | 0.10(0.30) | 0.13(0.04) | 0.09 | 0.12(0.41) |
| C (%) | 4.54(0.43) | 3.32(0.47) | 3.17(0.09) | 2.26 | 3.68(0.46) |
| CEC (cmo1^+^/kg) | 12.38(0.26) | 11.90(0.31) | 13.05(0.04) | 8.40 | 12.11(0.27) |
| K^+^ (cmo1^+^/kg) | 0.16(0.15) | 0.17(0.29) | 0.24(0.23) | 0.12 | 0.18(0.32) |
| Ca^2+^ (cmo1^+^/kg) | 0.38(0.28) | 0.38(0.40) | 0.44(0.57) | 0.21 | 0.40(0.39) |
| Mg^2+^ (cmo1^+^/kg) | 0.24(0.30) | 0.24(0.34) | 0.28(0.39) | 0.15 | 0.26(0.39) |
| Na^+^ (cmo1^+^/kg) | 0.08(0.16) | 0.10(0.29) | 0.13(0.12) | 0.05 | 0.10(0.26) |

**Table S6**. Primer sequences used for bacterial 16S rRNA (V4 region) and fungal ITS2 amplicon sequencing

| V4 region primer |  |
| --- | --- |
| 515F | 5′-GTGYCAGCMGCCGCGGTAA-3′ |
| 805R | 5′-GACTACHVGGGTATCTAATCC-3′ |
| ITS2 region primer |  |
| ITS86F | 5′-GTGAATCATCGAATCTTTGAA-3′ |
| ITS4 | 5′-TCCTCCGCTTATTGATATGC-3′ |

**Table S7**. Frequency information for each processing step in the bacterial 16S rRNA V4 and fungal ITS2.

| V4 region |  |  |  |  |
| --- | --- | --- | --- | --- |
| Step | Minimum | Median | Mean | Maximum |
| Original | 29,003 | 79,138 | 81,191 | 128,992 |
| Denoising | 20,763 | 48,036 | 47,999 | 71,928 |
| Rarefy | 20,542 | 20,542 | 20,542 | 20,542 |
| ITS2 region |  |  |  |  |
| Step | Minimum | Median | Mean | Maximum |
| Original | 62,577 | 74,312 | 78,991 | 178,780 |
| Denoising | 41,730 | 54,420 | 57,073 | 118,749 |
| Rarefy | 41,211 | 41,211 | 41,211 | 41,211 |

Table S8. Summary of statistical tests assessing the effect of plot area on species richness. Results are shown for a simple linear regression and a one-way analysis of variance (ANOVA) using plot area (m²) or plot-area class as predictors. Degrees of freedom (df; numerator, denominator) and the coefficient of determination (R²) are reported. Plot-area classes: 200, 225, 300, and 400 m². Including the control plots, there were 27 plots with an area of 200 m², and one plot each with areas of 225, 300, and 400 m².

| Analysis type | Predictor | Degrees of Freedom (num, den) | Test statistic | R² | p-value |
| --- | --- | --- | --- | --- | --- |
| Linear regression | Plot area (m²) | 1, 28 | F = 0.15 | 0.005 | 0.703 |
| One-way ANOVA | Plot area class^*^ | 3, 26 | F = 1.13 | – | 0.355 |

**Table S9**. Random forest hyperparameter testing status for identifying and evaluating the importance of indicator taxa that determine bacterial and fungal community differences among the four groups (Group1–3 & Decline). The evaluation was conducted using Out-of-Bag (OOB) with mtry and ntree. Refer to the main text for the definitions of Group1–3 and Decline groups.

| Fungi hyperparameter test | | | |
| --- | --- | --- | --- |
|  | mtry | ntree | OOB accuraacy |
| OOB18 | 5 | 1000 | 0.893 |
| OOB5 | 6 | 1300 | 0.867 |
| OOB2 | 6 | 800 | 0.840 |
| OOB16 | 6 | 1300 | 0.840 |
| OOB17 | 6 | 900 | 0.827 |
| OOB10 | 4 | 700 | 0.813 |
| OOB1 | 4 | 1200 | 0.800 |
| OOB15 | 5 | 1300 | 0.800 |
| OOB8 | 4 | 1000 | 0.787 |
| OOB19 | 3 | 1500 | 0.787 |
| OOB6 | 3 | 1300 | 0.760 |
| OOB12 | 3 | 800 | 0.760 |
| OOB11 | 3 | 1200 | 0.747 |
| OOB7 | 3 | 900 | 0.733 |
| OOB4 | 2 | 1400 | 0.720 |
| OOB3 | 1 | 800 | 0.547 |
| OOB14 | 1 | 1400 | 0.547 |
| OOB | 1 | 700 | 0.533 |
| OOB9 | 1 | 1500 | 0.520 |
| OOB13 | 1 | 800 | 0.493 |
| Bacteria hyperparameter test | | | |
| OOB18 | 5 | 1000 | 0.893 |
| OOB5 | 6 | 1300 | 0.867 |
| OOB2 | 6 | 800 | 0.840 |
| OOB16 | 6 | 1300 | 0.840 |
| OOB17 | 6 | 900 | 0.827 |
| OOB10 | 4 | 700 | 0.813 |
| OOB1 | 4 | 1200 | 0.800 |
| OOB15 | 5 | 1300 | 0.800 |
| OOB8 | 4 | 1000 | 0.787 |
| OOB19 | 3 | 1500 | 0.787 |
| OOB6 | 3 | 1300 | 0.760 |
| OOB12 | 3 | 800 | 0.760 |
| OOB11 | 3 | 1200 | 0.747 |
| OOB7 | 3 | 900 | 0.733 |
| OOB4 | 2 | 1400 | 0.720 |
| OOB3 | 1 | 800 | 0.547 |
| OOB14 | 1 | 1400 | 0.547 |
| OOB | 1 | 700 | 0.530 |
| OOB9 | 1 | 1500 | 0.520 |
| OOB13 | 1 | 800 | 0.493 |

**Table S10**. Results of Paired sample t-Test on soil chemical properties between multiple-tree mortality sites and healthy *P. densiflora* forests (Control). No statistically significant differences were observed in soil chemical properties between the two groups. SD indicates standard deviation. The abbreviations are as follows: total nitrogen (N), exchangeable cations (K⁺, Ca²⁺, Mg²⁺, and Na⁺), available phosphorus (measured as Olsen-P), organic carbon (C), organic matter (OM), cation exchange capacity (CEC), soil pH, and electrical conductivity (EC).

| Multiple-tree mortality sites vs control (Healthy *Pinus densiflora* forests) | | | | |
| --- | --- | --- | --- | --- |
| Division | | Descriptive Statistic | | t*(p)* |
|  |  | Average | SD |  |
| pH [1:5] | Multiple-tree mortality | 4.253 | 0.428 | –1.567(0.139) |
|  | Control | 4.431 | 0.333 |  |
| EC (dS/m) | Multiple-tree mortality y | 0.079 | 0.042 | 2.109(0.053) |
|  | Control | 0.064 | 0.030 |  |
| OM (%) | Multiple-tree mortality | 5.545 | 4.948 | 0.846(0.412) |
|  | Control | 1.998 | 1.505 |  |
| P_2_O_5_ (mg/kg) | Multiple-tree mortality | 20.064 | 5.644 | 2.098(0.055) |
|  | Control | 17.511 | 4.579 |  |
| N (%) | Multiple-tree mortality | 0.115 | 0.047 | 1.167(0.263) |
|  | Control | 0.098 | 0.033 |  |
| C (%) | Multiple-tree mortality | 3.676 | 1.673 | 1.285(0.220) |
|  | Control | 2.995 | 0.841 |  |
| CEC (cmol^+^/kg) | Multiple-tree mortality | 12.113 | 3.218 | 1.131(0.277) |
|  | Control | 10.913 | 2.300 |  |
| K^+^ (cmol^+^/kg) | Multiple-tree mortality | 0.185 | 0.060 | –0.097(0.924) |
|  | Control | 0.188 | 0.077 |  |
| Ca^2+^ (cmol^+^/kg) | Multiple-tree mortality | 0.400 | 0.156 | –1.683(0.115) |
|  | Control | 0.695 | 0.651 |  |
| Mg^2+^ (cmol^+^/kg) | Multiple-tree mortality | 0.264 | 0.103 | –1.099(0.290) |
|  | Control | 0.339 | 0.223 |  |
| Na^+^ (cmol^+^/kg) | Multiple-tree mortality | 0.100 | 0.027 | –1.090(0.294) |
|  | Control | 0.123 | 0.084 |  |
